# UPLC-ESI-QTRAP-MS-Based Metabolomics Revealed Changes in Biostimulant-Related Metabolite Profiles in *Zingiber mioga* Flower Buds During Development

**DOI:** 10.3390/metabo15060358

**Published:** 2025-05-28

**Authors:** Jiao Xie, Yahan Zhou, Zhifei Cheng, Huijuan Liu

**Affiliations:** 1 The key Laboratory of Environmental Pollution Monitoring and Disease Control, Ministry of Education, School of Public Health, Guizhou Medical University, Guiyang 561113, China; xiejiao@gmc.edu.cn; 2 School of Light Industry Science and Engineering, Beijing Technology and Business University, Beijing 100048, China; zhouyahan@btbu.edu.cn; 3 Basic Teaching Department, Guizhou Vocational College of Agriculture, Qingzhen 551499, China; 18786767768@139.com

**Keywords:** *Z. mioga* flower buds, growth and development, different stages, biostimulants

## Abstract

**Background:** The composition and abundance of biostimulants are important factors in the formation of the flavour and nutritional value of flower buds, as well as key factors influencing their growth and development. **Methods:** Therefore, the variation characteristics of phenolic acids, nucleotides and derivatives, alkaloids, lipids, tannins, terpenoids and others in *Z. mioga* flower buds during the growth and development were studied by UPLC-MS/MS. **Results:** The vast majority of the 204 compounds identified in this study showed a clear upward trend throughout the bud development, accumulating to a maximum at maturity. Considering both the PCA and HCA results, the four growth stages were effectively separated, indicating the significant differences between the stages, and the late developmental stage (SG3) was likely to be the key node in growing and developing flower buds. Differential metabolites that affected the stage division were screened by OPLS-DA. **Conclusions:** Correlation analysis based on the key top 50 differential metabolites showed that biostimulant-related compounds collectively influenced the growth and maturation of *Z. mioga* flower buds in a joint and comprehensive manner.

## 1. Introduction

*Zingiber mioga* Roscoe (*Z. mioga*), also known as wild ginger, alpine ginger or monkey ginger, belongs to the ginger family and the ginger genus, and is a perennial vegetable with medicine and food uses [1]. The flower buds of *Z. mioga*, which develop from the underground rhizome, are the edible part of the plant, and the mature buds exposed to the ground are shaped like a purple-red pinecone [2,3,4]. In recent years, the edible *Z. mioga* flower bud, which is rich in nutrients such as sugars and fatty acids, and bioactive compounds such as anthocyanins and proanthocyanidins, have aroused great interest with the rapid growth of the national economy and the gradual deepening of people’s understanding of the relationship between nutrition and health. Previous studies have shown that sufficient growth is crucial for increasing the value of *Z. mioga* flower buds. The composition and abundance of the nutrients and bioactive compounds that influence the edible and medicinal value of the buds show obvious temporal distribution characteristics, i.e., obvious stage dependence [3,4,5].

Biostimulants are plant-derived substances that can control plant growth and development, promote germination and flowering, boost plant resistance to stress and disease and improve nutrient absorption [6,7,8]. Several types of compounds have been shown to be biostimulatory, including sugars, lipids, amino acids, nucleotides, phenolic acids, tannins, terpenoids and alkaloids, in addition to the well-known plant hormones and growth regulators such as auxin, ethylene and abscisic acid [7,9,10]. Previous studies have shown that the growth of flower buds is regulated by endogenous hormones, such as indole-3-acetic acid and cytokinin, which typical promote growth and engage in complex interactions to regulate this process [11,12]. Endogenous terpenoids play a positive role in plant bud growth through antibacterial properties, regulating plant growth and development and improving plant adaptability to the environment [13]. Endogenous lipids can influence the growth and development of plant buds by regulating the levels and activity of plant hormones and the process of plant organogenesis. Existing studies have suggested an interactive dialogue between the florigen flowering LOCUS T (FT) and phosphatidylglycerol (PG) in lipids, both of which are involved in regulating the flowering process in plants [14]. Endogenous nucleotides and their derivatives can indirectly participate in the regulation of plant bud growth and development by promoting cell division and regulating hormone levels and the influence of gene expression [7,15,16]. Several studies have confirmed the importance of phenolic acids in the differentiation of plant flower buds. For example, the up-regulation of chlorogenic acid is involved in stress-induced flowering, and the accumulation of phenolic acids changes significantly as flower buds grow and develop [17,18]. Alkaloids affect the growth and development of plants by promoting or inhibiting certain physiological processes. This affects the growth and development of flower buds [19]. Therefore, the biostimulants listed above play an indispensable role in growing and developing flower buds. In addition, it should be emphasized that the composition of biostimulant-related metabolites in flower buds is extremely diverse, and the relationships between different types of compounds are complicated, making it difficult to attribute their effects on flower bud development to a single compound or class of compounds.

Although biostimulants can control plant growth and development, as well as promote germination and flowering, the correlation between specific biostimulants and the developmental stage of *Z. mioga* flower buds is unclear. This is important when considering the use of biostimulants to improve the quality of *Z. mioga* flower buds. At present, ultra-performance liquid chromatography-electrospray ionization-triple quadrupole-linear ion trap mass spectrometry (UPLC-ESI-Qtrap-MS/MS) is a common method of detecting metabolites in Zingiberaceae, such as *Alpinia hainanensis* and *Z. mioga* [4,20]. Therefore, the temporal variation characteristics of biostimulant-related metabolites (including phenolic acids, nucleotides and derivatives, alkaloids, lipids, tannins, terpenoids and other metabolites) in the *Z. mioga* flower buds at different growth stages were determined by UPLC-ESI-Qtrap-MS/MS and their correlations, with the primary objective of providing a reference for the relationship between endogenous biostimulants in *Z. mioga* flower buds and their quality formation during the developmental stages, as well as providing the guidance for exploring new plant biostimulants.

## 2. Materials and Methods

### 2.1. Plant Material

**Figure 1:**
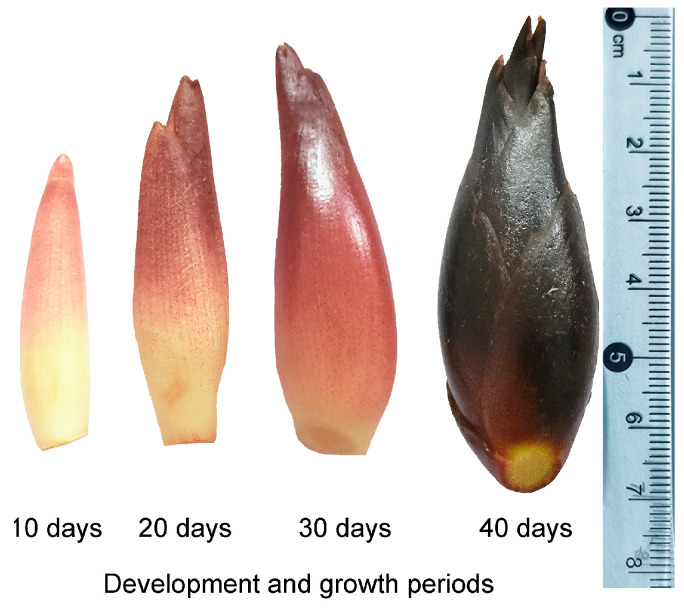
Schematic representation of the flower buds of *Z. mioga* during development.

The *Z. mioga* flower bud samples used in this study were collected in 2020 from Congjiang County [latitude 25°50′33.8″ (N), longitude 109°7′18.3″ (E)] in Guizhou Province, China, and were certified by experts; their appearance in Figure 1 is consistent with the literature [4,21]. Previous studies have confirmed that the period from flower bud formation to maturity is 40 days, and its growth cycle is artificially divided into four stages according to the appearance characteristics and nutrient accumulation characteristics of the buds [3,4,5,21,22]. In this study, SG1, SG2, SG3 and SG4 referred to 10, 20, 30 and 40 days of growth, respectively, which could also be referred to as the germination, budding, late development and maturity stages. Fifteen plants were selected at random, and a total of 45 flower buds were collected at each developmental stage. Therefore, there were 15 flower buds per biological replicate at each developmental stage. The standard for sample collection was that the flower buds derived from the same growth stage should be highly consistent in appearance and development time, and that there should be no objective interfering factors such as mechanical damage, pests or diseases. Samples collected in the field were transferred to the laboratory in time, gently cleaned of soil and debris adhering to the bud surface and dried naturally, then frozen in liquid nitrogen and transferred to the −80 °C refrigerator until sample extraction.

### 2.2. Chemicals and Reagents

The chemical reagents used for the analysis of phenolic acids, nucleotides and derivatives, alkaloids, lipids, tannins and other metabolites were all HPLC grade. Methanol and acetonitrile were of chromatographic grade and were supplied by Merck Co (Darmstadt, Germany). The chromatographic grade standards of the 204 compounds listed in Tables S1 and S2, and the internal standard of D3-leucine were all supplied by BioBioPha/WAKO/Sigma Aldrich (St Louis, MO, USA).

### 2.3. Extraction of Metabolites Related to Biostimulants

The method for extracting phenolic acids, nucleotides and derivatives, alkaloids, lipids, tannins and their biostimulants in *Z. mioga* flower buds was based on the study by Wei et al. (2023) [4]. First, the collected *Z. mioga* flower buds were subjected to vacuum freeze-drying. The dried flower bud samples were ground to fine powder using a ball mill instrument in an environment filled with liquid nitrogen, with parameters of 30 Hz and 1.5 min. A total of 1.2 mL of 70% methanol was used as the extraction solution to extract 100 mg of accurately weighed powder. All of the extraction systems were vortexed for 30 s every 30 min, repeated for 6 times, and then the treated systems were refrigerated overnight at 4 °C. The extraction system, which was left overnight, was centrifuged at 4 °C and 12,000 rpm for 10 min, and then the supernatant was carefully aspirated and filtered using 0.22 µm microporous membranes. Finally, the extracts obtained were placed in vials for subsequent analysis.

### 2.4. Analysis of Metabolites Related to Biostimulants

The filtered extracts containing phenolic acids, nucleotides and derivatives, alkaloids, lipids, tannins and other biostimulants were subjected to UPLC-MS/MS analysis, and the experiment was repeated three times for each stage. The data acquisition system was UPLC-ESI-Q TRAP-MS/MS, which was a qualitative and quantitative instrument consisting of two main components: UPLC (Shimadzu Nexera X2, Shimadzu Corporation, , Kyoto, Japan) and MS/MS (SCIEX 4500 QTRAP, Applied Biosystems, Framingham, MA, USA). Analyst 1.6.3 software (AB Sciex) operated both positive and negative ion modes on a triple quadrupole linear ion trap (Q TRAP) mass spectrometer, and the AB4500 Q TRAP UPLC/MS/MS system with an ESI turbine-ion spray interface installed performed both LIT and triple quadrupole (QQQ) scans to complete an accurate quantitative analysis of compounds in the samples.

The specific parameters and operations of the chromatography were as follows: UPLC was installed with an SB-C18 column (2.1 mm × 100 mm, 1.8 µm). The system flow rate and column temperature were set and maintained at 0.35 mL/min and 40 °C, respectively, and the injection volume was precisely limited to 4 μL. The solvent system consisted of eluent A and eluent B, which were ultrapure water with 0.1% formic acid and acetonitrile with 0.1% formic acid, respectively. The elution procedure was set up as follows: First, the composition ratio of 5% eluent B and 95% eluent A was the initial condition of the gradient elution. The amount of eluent A was then linearly reduced to 5% and the amount of eluent B was increased to 95% within 9 min and maintained for 1 min. Subsequently, the proportion of eluent B was rapidly reduced to 5% in the following 1 min and balanced under this condition for 14 min.

The specific parameters and operations of mass spectrometry were as follows: the source temperature of the ion source was adjusted and stabilised at 550 °C; the ion spray voltage (IS) of turbine spray varied with different ion modes, which were 5500 V and −4500 V in positive and negative ion modes, respectively. Collision-activated dissociation (CAD) was set to high mode to improve the sensitivity and selectivity of the mass spectrometry analysis, and the ion source gas I and II (GSI and GSII) and curtain gas (CUR) were selectively set to 50, 60 and 25.0 psi, respectively. The tuning and calibration of the system were carried out using 10 and 100 μmol·L^−1^ solutions of polypropylene glycol in QQQ and LIT modes, respectively. The QQQ scan was performed in multiple reaction monitoring (MRM) mode with nitrogen selected as the collision gas and the parameter set to medium. The key parameters of each MRM ion pair were obtained by optimizing the declustering potential (DP) and collision energy (CE). The characteristic ions of each metabolite were screened using a triple quadrupole. The signal intensity of the characteristic ions was obtained using a detector. The sample’s downstream mass spectrometry file was opened using MultiaQuant software to integrate the chromatographic peaks and perform corrections. The quality control sample was prepared by mixing sample extracts and was used to analyse the repeatability of the sample under the same processing method. During the analysis process, a quality control sample was inserted every 10 monitoring and analysis samples to monitor the repeatability and applicability of the analysis process. As metabolites were eluted at different times, specific ion pairs corresponding to these metabolites were detected, and the identification of these metabolites was performed using the Metabolite Information Public Database (Lianchong Bio’s own database, LC Sciences, Hangzhou, China). The final relative content of each identified metabolite depended on the ratio of the peak area of the metabolite to the peak area of the internal standard D3-leucine, where the concentration of the internal standard was 5 mg·L^−1^. For subsequent statistical analyses, the relative amounts of each metabolite obtained were used.

### 2.5. Statistical Analysis

In this study, IBM SPSS Statistics 22.0, GraphPad Prism 7 and Photoshop 6.0 were used for the statistical processing and graphic drawing of all data. The application of R software (R i386 ver. 3.3.3) contributed to the effective completion of principal component analysis (PCA). The relative content of each metabolite was expressed as the mean ± standard deviation, and Duncan’s multiple comparisons were performed to show the differences in the relative content of each metabolite in *Z. mioga* flower buds at different developmental stages. The identification of the differential metabolites, which fulfilled the conditions of VIP > 1 and *p* < 0.05, was carried out by OPLS-DA. An analysis of the correlation between different growth stages and different metabolites was performed using the R software package (R version 3.6.3), with the type and degree of correlation determined by the Pearson correlation coefficient. Statistical significance was determined using *p* < 0.05 as the criterion.

## 3. Results and Discussion

### 3.1. Identification and Quantitative Descriptive Analysis of Biostimulant-Related Metabolites

The UPLC-ESI-Q TRAP-MS/MS method was used to identify the metabolites of phenolic acids, nucleotides and derivatives, alkaloids, lipids, tannins and others in the flower buds of *Z. mioga* during the whole growth and development period. The MRM of the metabolites in *Z. mioga* flower buds was shown in Supplementary Figure S1. According to the chemical identification characteristics of the standard compounds, the related information of parent ions, characteristic fragment ions and the molecular weight of each compound were obtained (Tables S1 and S2). In this study, 204 metabolites, including fifty-three phenolic acids, forty-seven nucleotides and derivatives, twenty-eight alkaloids, forty-three lipids, two tannins, seven terpenoids and twenty-four other compounds, were successfully identified from *Z. mioga* flower buds at different growth stages. Of these, the identified phenolic acids, nucleotides and derivatives, alkaloids and the other compounds (including vitamins and phytohormones) were consistent with the findings of Wang et al. (2024) regarding the compounds present in *Zingiber striolatum* Diels [23]. Overall, most of the 204 metabolites belonging to seven compound types showed similar dynamic characteristics related to change during the growth and development of the flower buds; that is, the relative content of metabolites accumulated with the increase in the developmental degree of the flower buds. The highest levels of metabolites were found at 30 or 40 days of growth (Tables S1 and S2). For the above seven types of compounds found in *Z. mioga* flower buds, the relative content of most metabolites varied among the four different growth stages, with the most significant difference observed between the last two stages. At the maturity stage, the best harvesting stage recommended by Wei et al. (2023) and Liu et al. (2024) [3,4], the metabolites with the highest relative abundance were 2′-deoxyguanosine, LysoPC 16:1 and choline, followed by n-benzylmethylene isomethylamine, sinapoylglucuronic acid, 6-deoxyfagomine and desoxyinosin-5′-phosphat, ranging from 0.19 to 7.87, 1.03 to 6.89, 3.16 to 6.41, 2.32 to 4.18, 0.56 to 2.53, 1.76 to 2.51 and 1.66 to 2.51, respectively. Studies have shown that endogenous nucleotides and their derivatives, as well as lipids, can affect the growth and development of flower buds by regulating phytohormone levels, either directly or indirectly [7,14,15,16]. This study found that the metabolites with the highest relative abundance at maturity were 2′-deoxyguanosine and LysoPC 16:1, suggesting that these two classes of compounds may regulate the maturity of *Z. mioga* directly or indirectly by controlling phytohormones. In addition, adenylthiomethylpentose, adenine, adenosine, thymidine, piperidine, 4-hydroxymandelonitrile, LysoPC 16:0, LysoPC 18:1, LysoPE 18:2 and LysoPC 15:1, with relative content ranging from 1.0 to 2.1, were also major contributors to the metabolite profiles of *Z. mioga* flower buds, which were at the intermediate level of relative abundance. The above 17 compounds that contributed most significantly to dynamic changes in the metabolite profiles of *Z. mioga* flower buds during growth and development included one phenolic acid, six nucleotides and their derivatives, six alkaloids and five lipids. The relative abundances of thirteen phenolic acids (including 1-*O*-glucosyl sinapate, koaburaside, [10]-paradol, caffeic acid, coniferin, 2-feruloyl-sn-glycerol, Di-*O*-glucosylquinic acid, syringaldehyde, 1-*O*-feruloyl-d-glucose, brevifolincarboxylic acid, 5-*O*-feruloylquinic acid, benzamide and phloretic acid), four nucleotides and derivatives (including uridine 5′-monophosphate, triphosphopyridine nucleotide, 2′-deoxycytidine-5′-monophosphate and 7-methylguanine), two alkaloids (including 10-formyltetrahydrofuran and indole-3-acetic acid), two lipids (including 1-*O*-feruloyl-3-*O*-caffeoylglycerol and 2-linoleoylglycerol-1-*O*-glucoside), and five other compounds (including scopoletin, ligraminol B, gingerenone A, officinarumane C and creatine) were the lowest at the maturity stage, and the contribution of the above 26 compounds to the metabolite profiles of the other three growth stages was also meagre. The existence of individual cases was also worth noting; according to the semi-quantitative results, it was found that six phenolic acids (salicylic acid, brevifolincarboxylic acid, 5-*O*-feruloylquinic acid, benzamide, [4]-gingerdiol and phloretic acid), twelve nucleotides and their derivatives (guanosine 3′,5′-cyclic monophosphate, 2′-deoxyadenosine, 2-aminopurine, cordycepin DL-2-aminoadipic acid, 2′-deoxycytidine, 5-methylcytosine, hypoxanthine, 2′-deoxycytidine-5′-monophosphate, allopurinol, 2′-deoxyuridine, uric acid and 7-methylguanine), four alkaloids (*N′, N″, N‴*-p-coumaroyl-cinnamoyl-caffeoyl spermidine, indole-3-carboxylic acid, *N*-oleoylethanolamine and indole-3-acetic acid) and six lipids (1-linoleoylglycerol, LysoPC 20:3, LysoPE 16:1, LysoPE 16:0, LysoPE 17:1 and 2-linoleoylglycerol-1-*O*-glucoside) were not detected at the germination stage, even at the first three stages, indicating that the production, accumulation and release of these 28 metabolites occurred mainly at the maturity stage of flower buds. In summary, these results suggested that adequate growth could increase the diversity and overall abundance of metabolites in *Z. mioga* flower buds, which largely determine the flavour and nutritional value of this wild vegetable with regional characteristics. Therefore, the composition and abundance of metabolites in *Z. mioga* flower buds was primarily dependant on their growth and maturity levels, which was consistent with the findings of Liu et al. (2024) and Wei et al. (2023) [3,4].

### 3.2. Analysis Profiles of Biostimulant-Related Metabolites

**Figure 2:**
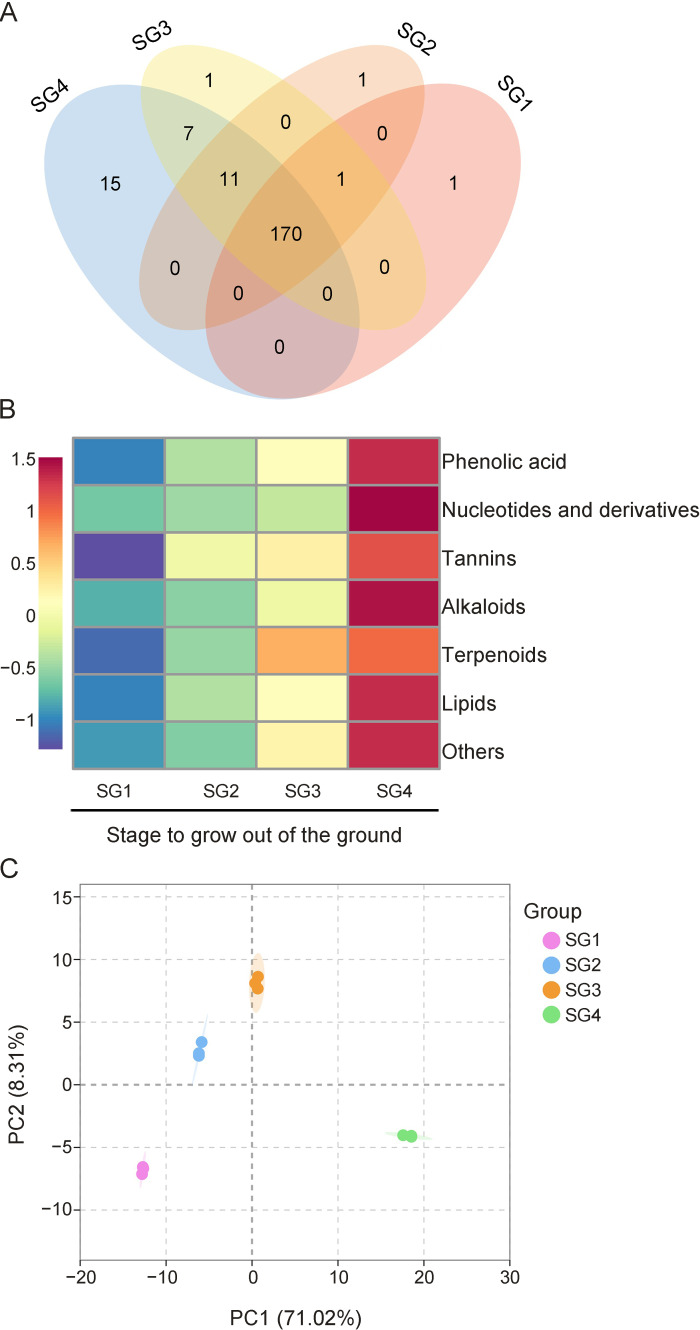
Types and multivariate statistical analysis of metabolites in the flower buds of *Z. mioga* at different development stages (SG1, SG2, SG3 and SG4 stage). (**A**), the Venn diagram; (**B**), the classification and relative content of metabolites; and (**C**), the principal component analysis (PCA).

*Z. mioga* flower buds have high nutritional value and unique flavour, which is related to the rich composition of metabolites and the complex mixture of metabolites belonging to different chemical categories. In this study, further analysis was performed on the 204 important metabolites identified from *Z. mioga* flower buds. Figure 2A clearly showed the number of unique and common metabolites at each growth stage. Obviously, 170 metabolites coexisted in four different growth stages, and the number of unique metabolites in the mature stage was significantly higher than that in the other three stages, which was also reflected in Tables S1 and S2. To analyse from the perspective of “metabolite type”, a cluster analysis was performed on the data obtained. As shown in Figure 2B, Tables S1 and S2, the 204 identified metabolites were divided into phenolic acids (53), nucleotides and derivatives (47), alkaloids (28), lipids (43), tannins (2), terpenoids (7) and other compounds (24). In terms of the number of metabolites in each compound type, the largest chemical group was phenolic acids, followed by nucleotides and derivatives, and lipids. In addition, the abundance of seven types of metabolites was the lowest at the early growth stage (Figure 2B). In addition, the seven types of metabolites reached the maximum at the maturity stage (Figure 2B), which was consistent with the expression patterns of sugars, organic acids, fatty acids, amino acids and volatile compounds during the growth and development of flower buds [3,4]. In order to further understand the effects of growth and development on the above seven types of metabolites in *Z. mioga* flower buds, multivariate statistical analysis was performed on the 204 metabolites obtained by semi-quantitative analysis, and the detailed information is shown in Figure 2 and Supplementary Figure S2. Taking into account the complexity of the data, the implementation of principal component analysis (PCA) allowed the visual representation of the distribution of the data [24]. The 204 metabolites belonging to the seven compound types exhibited significant changes throughout the entire growth and development period, as illustrated by Figure 2C. The 12 samples from the four different growth stages on the PCA score plot showed the characteristics of a high aggregation of sample points within the group and obvious separation of sample points between groups, indicating that the flower buds at different growth stages could be well separated by the first two principal components, and the contribution level of the two principal components to the total variability was 79.33%, with PC1 and PC2 accounting for 71.02% and 8.31%, respectively (Figure 2C). Furthermore, the use of hierarchical cluster analysis (HCA) intuitively represented the cluster structure of the samples and the hierarchical structure of the sample distribution [25]. As described in Supplementary Figure S3, 12 samples from four different growth stages were grouped into three categories by hierarchical cluster analysis, of which SG2 and SG3 were classified as one category, while SG1 and SG4 belonged to another. The results of PCA and HCA were mutually confirmed, the metabolite profiles in *Z. mioga* flower buds were closely related to the maturity degree of the flower buds, and it was speculated that the above seven types of compounds underwent a dramatic change at the SG3 and SG4 stages.

### 3.3. OPLS-DA Results

**Figure 3:**
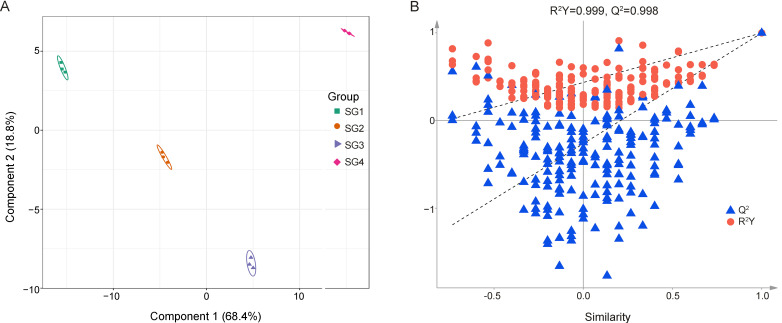
The OPLS-DA results. (**A**) indicates the OPLS-DA plot scores; (**B**) shows the S-plot of OPLS-DA obtained by permutation testing. OPLS-DA, orthogonal partial least squares discriminant analysis.

In order to ensure that the analysis of non-orthogonal variables and the analysis of orthogonal variables do not interfere with each other, so as to obtain reliable information on the intergroup differences in metabolites and the degree of correlation between groups, the implementation of OPLS-DA is essential, as it can filter out the orthogonal variables in metabolites that are not related to the categorical variables [26]. Figure 3A showed the OPLS-DA score chart of four different growth and development stages of *Z. mioga* flower buds, and the score chart used the score value of the principal component derived from the orthogonal signal correction as the abscissa. Obviously, from the point of view of the abscissa alone, the differences in the four different stages were extremely significant. Specifically, the 12 sample points derived from the four groups were effectively distributed within the confidence interval, and the aggregation of points within the group and the separation of points between the groups meant that the four developmental stages were well distinguished and the differences were significant (Figure 3A). The above information once again explained the importance of the degree of growth and development on the composition and abundance of metabolites in *Z. mioga* flower buds [3,4]. In addition, SG1, SG2 and SG3 were placed in a row, and the three of them were all far away from SG4. Therefore, the late developmental stage (SG3) seemed to be the crucial turning point for the growth and development of flower buds. To prevent the overfitting of the OPLS-DA model, the permutation test method was used to validate the OPLS-DA model (Figure 3A), with the model fit evaluated over 200 iterations in this study. The cumulative values of R^2^Y and Q^2^ were 0.999 and 0.998, respectively, and the corresponding *p* values of Perm R^2^Y and Perm Q^2^ were all less than 0.005, strongly confirming that the OPLS-DA model was reliable and well fitted, and that there was no overfitting problem.

### 3.4. Identification and K-Means Cluster Analysis of Differential Metabolites

**Figure 4:**
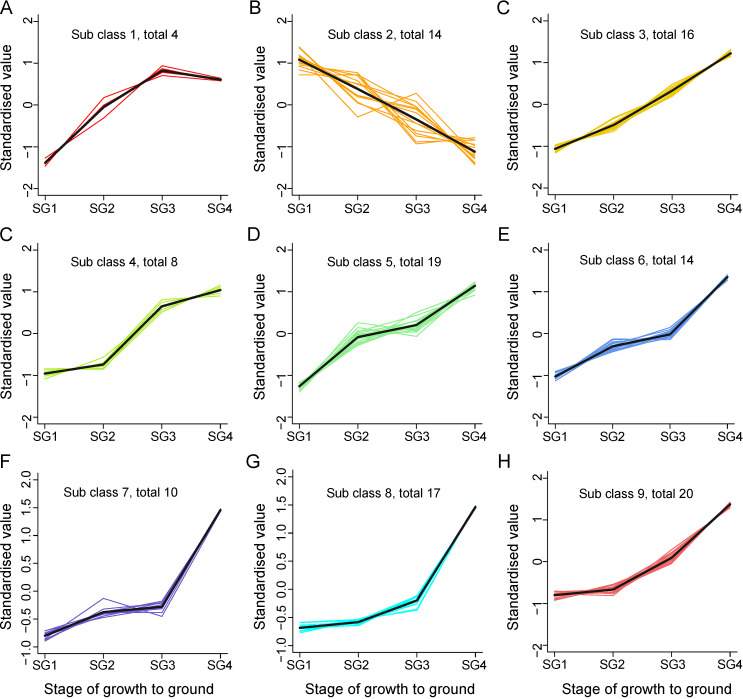
K-means plot of the relative contents of differential metabolites in the flower buds of *Z. mioga* at the developing stage. The horizontal line represents the developing stage; the vertical line indicates the relative content of the standardised metabolites; subclass represents the number of metabolite classes with the same trend of change; and total represents the number of metabolites in that category. SG1, 10 days of growth or the germination stage; SG2, 20 days of growth or the budding stage; SG3, 30 days of growth or the late developmental stage; and SG4, 40 days of growth or the maturity stage.

The identification and determination of differential metabolites through the variable importance in projection (VIP) value was very important to measure the strength and explanatory power of each metabolite expression pattern on the categorical discrimination of each group of samples, and the supervised discriminant analysis performed under the OPLS-DA statistical method effectively solved this problem [3,27]. The differential metabolites identified by combining the *p* value derived from the t-test with the VIP value derived from the first principal component of OPLS-DA exhibited the characteristic that the greater the VIP value, the greater the contribution to the separation between sample groups. A total of 122 differential metabolites were identified from *Z. mioga* flower buds at different growth stages, and most of the metabolites were up-regulated with increasing maturity (Figure 4 and Table S3). In order to present the temporal expression patterns of the above differential metabolites at different stages of growth and development in a more intuitive way, the K-means clustering analysis (K-means) was performed and the completion of this analysis required the standardization and centralization of the relative content of all differential metabolites. According to the characteristics of the expression patterns throughout the growth cycle, 122 differential metabolites were grouped into nine classes, and classes 1–9 included four, fourteen, sixteen, eight, nineteen, fourteen, ten, seventeen and twenty metabolites, respectively (Figure 4). As shown in Figure 4, the accumulation of 104 metabolites (including sixteen alkaloids, thirty-six lipids, twenty-three nucleotides and derivatives, eighteen phenolic acids, one tannin, two terpenoids and eight others) in class 3, class 4, class 5, class 6, class 7, class 8 and class 9 increased with the improvement of growth and development level. It should be noted that the differential metabolites belonging to class 4–class 9 showed different accumulation rates at different stages of growth and development, and the tortuous rising lines reflected this feature. Meanwhile, cluster 1 contained four different metabolites (including one alkaloid, one phenolic acid, one terpenoid and one other compound), which showed a trend of increasing first and then decreasing throughout the whole developmental period, with SG3 being the crucial turning point. Of these, it is noteworthy that galanal A was the one other compound. This differential metabolite was the major contributor to the unique spicy flavour of *Z. mioga* flower buds, previously reported only in this plant [2,3]. The 14 differential metabolites (including three alkaloids, one lipid, one nucleotide and derivative, five phenolic acids, one terpenoid and three others) in cluster 2 were special, and their abundance decreased sharply from germination to maturity, reaching the lowest value at maturity.

### 3.5. Systematic Analysis of Key Differential Metabolites

**Figure 5:**
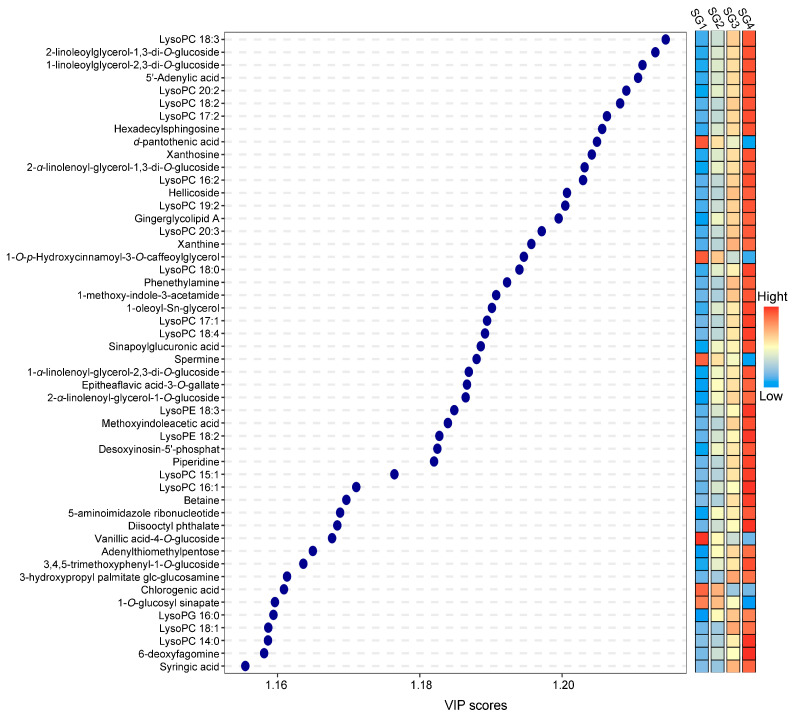
The top 50 differential metabolites with the highest VIP values during growth and development derived from the OPLS-DA method. VIP, variable importance in projection; OPLS-DA, orthogonal partial least squares discriminant analysis. Both the VIP value > 1 and *p* < 0.05 were considered as significant differences. SG1, 10 days of growth or the germination stage; SG2, 20 days of growth or the budding stage; SG3, 30 days of growth or the late developmental stage; and SG4, 40 days of growth or the maturity stage.

The greater the VIP value of the differential metabolite, the greater its contribution to stage partitioning. As shown in Figure 5, the top 50 differential metabolites with the highest VIP values were extracted from four different developmental stages, consisting of eight alkaloids (phenethylamine, 1-methoxy-indole-3-acetamide, spermine, methoxyindoleacetic acid, piperidine, betaine, 3-hydroxypropyl palmitate glc-glucosamine and 6-deoxyfagomine), twenty-six lipids (LysoPC 18:3, 2-linoleoylglycerol-1,3-di-*O*-glucoside, 1-linoleoylglycerol-2,3-di-*O*-glucoside, LysoPC 20:2, LysoPC 18:2, LysoPC 17:2, hexadecylsphingosine, 2-*α*-linolenoyl-glycerol-1,3-di-*O*-glucoside, LysoPC 16:2, LysoPC 19:2, gingerglycolipid A, LysoPC 20:3, 1-*O*-p-hydroxycinnamoyl-3-*O*-caffeoylglycerol, LysoPC 18:0, 1-oleoyl-Sn-glycerol, LysoPC 17:1, LysoPC 18:4, 1-*α*-linolenoyl-glycerol-2,3-di-*O*-glucoside, 2-*α*-linolenoyl-glycerol-1-*O*-glucoside, LysoPE 18:3, LysoPE 18:2, LysoPC 15:1, LysoPC 16:1, LysoPG 16:0, LysoPC 18:1 and LysoPC 14:0), six nucleotides and derivatives (5′-adenylic acid, xanthosine, xanthine, desoxyinosin-5′-phosphat, 5-aminoimidazole ribonucleotide and adenylthiomethylpentose), seven phenolic acids (sinapoylglucuronic acid, diisooctyl phthalate, vanillic acid-4-*O*-glucoside, 3,4,5-trimethoxyphenyl-1-*O*-glucoside, chlorogenic acid, 1-*O*-glucosyl sinapate and syringic acid), one tannin (epitheaflavic acid-3-*O*-gallate), and two others (*d*-pantothenic acid and hellicoside). Obviously, in terms of the number of metabolites, lipids accounted for the largest proportion, followed by alkaloids and phenolic acids (Supplementary Figure S3). Moreover, Figure 5 also provided a heat map of the top 50 differential metabolites that played an important role during the growth and development of *Z. mioga* flower buds, which clearly and intuitively showed the temporal variation characteristics of these important differential metabolites throughout the flower bud development period. Specifically, most compounds accumulated continuously as growth and development progressed, reaching the maximum abundance at the maturity stage, whereas the trends for d-pantothenic acid, 1-*O*-*p*-hydroxycinnamoyl-3-*O*-caffeoylglycerol, spermine, vanillic acid-4-*O*-glucoside, chlorogenic acid and 1-*O*-glucosyl sinapate were the opposite. In summary, the above information, together with the results of PCA, HCA and K-means analysis, strongly confirmed the importance of growth and development in determining the composition and abundance of metabolite profiles in *Z. mioga* flower buds. This finding was consistent with the research of Liu et al. (2024) and Wei et al. (2023) [3,4].

### 3.6. Correlation Analysis of Different Stages and Differential Metabolites

**Figure 6:**
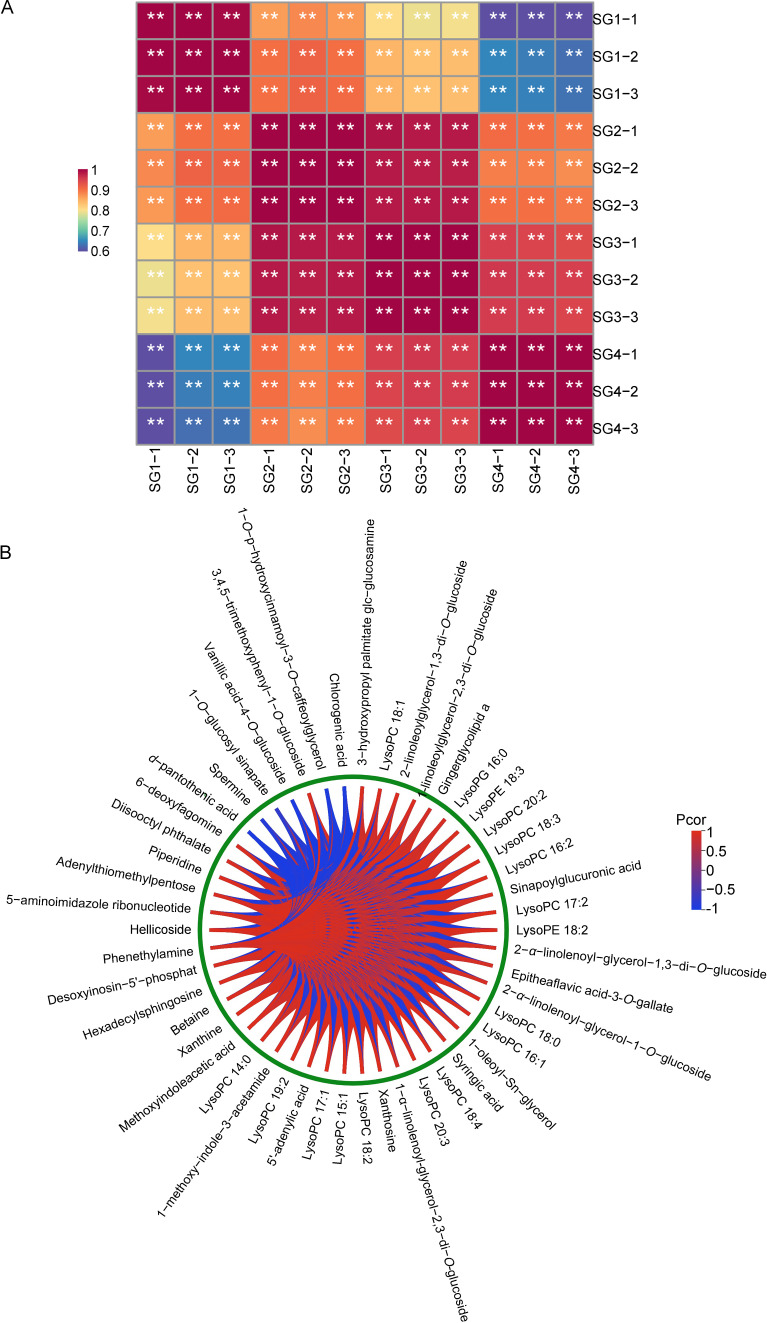
Correlation analysis based on the top 50 differential metabolites in the flower buds of *Z. mioga* at the developmental stage with the highest VIP value. (**A**), the correlation heatmap for each developmental stage, where *p* < 0.01 was considered as a significant difference and marked as ** based on the Duncan’s multiple comparisons; (**B**), the chord graph referring to the top 50 differential metabolites of *Z. mioga* with the highest VIP values. SG1-1/SG1-2/SG1-3, the replications for 10 days of growth or the germination stage; SG2-1/SG2-2/SG2-3, the replications for 20 days of growth or the budding stage; SG3-1/SG3-2/SG3-3, the replications for 30 days of growth or the late developmental stage; and SG4-1/SG4-2/SG4-3, the replications for 40 days of growth or the maturity stage.

In order to confirm the rationality of the division of the four growth stages of *Z. mioga* flower buds and to visualise the correlation between the stages, the cluster heat map analysis was performed and the cross-talk between the top 50 differential metabolites that played an important role in stage division was explored (Figure 6). As shown in Figure 6A, the correlation coefficients for the four different growth stages were all greater than 0.6. In addition, the three samples at the same stage were very similar. Obviously, SG3, the late developmental stage, was the key period for the synthesis and accumulation of phenolic acids, nucleotides and derivatives, tannins, alkaloids, terpenoids and lipids in the metabolic profile of *Z. mioga* flower buds. This finding corresponded with the differential expression patterns of sugars, organic acids, fatty acids, amino acids and volatiles in *Z. mioga* flower buds at different development stages, i.e., this again demonstrated the importance of sufficient growth, particularly at the SG3 stage, for the formation of nutrition and flavour formation in *Z. mioga* flower buds [3,4]. To further explore the crucial role of the key differential metabolites in stage division, as well as how they coordinated with each other to affect flower bud growth and development, a chord diagram was constructed to clearly and simply show the complex cross-dialogue relationship between these metabolites (Figure 6B). Interestingly, we found that the correlation between the top 50 key differential metabolites was significant, emphasizing that these metabolites coordinated and jointly regulated the growth and development of *Z. mioga* flower buds. In particular, as shown in Figure 6B, there were some interesting correlations between the six types of metabolites (eight alkaloids, twenty-six lipids, six nucleotides and derivatives, seven phenolic acids, one tannin and two others). For example, *d*-pantothenic acid was significantly negatively correlated with the remaining 49 metabolites, and spermine, 1-*O*-glucosyl sinapate, vanillic acid-4-*O*-glucoside, 1-*O*-p-hydroxycinnamoyl-3-*O*-caffeoylglycerol and chlorogenic acid were all mainly negatively correlated with the remaining compounds. The metabolism of certain fatty acids and phospholipids requires coenzyme A, which is synthesised using *d*-pantothenic acid. Consequently, the metabolism of these fatty acids uses up a significant amount of coenzyme A, thereby accelerating the conversion of *d*-pantothenic acid into coenzyme A [28]. This may explain the negative correlation between pantothenic acid and these fatty acids. In addition to the six metabolites mentioned above, the other 44 compounds were all mainly positively correlated with other compounds. Thus, the biostimulant-related compounds alkaloids, lipids, nucleotides and their derivatives, phenolic acids, tannins and other compounds collectively influenced the growth and development of *Z. mioga* buds through mutual synergistic effects, especially their growth up to the GS3 stage. Among these, alkaloids, lipids, nucleotides and their derivatives, phenolic acids and tannins might identify as biostimulants that regulate the growth and development of *Z. mioga* buds. This has been demonstrated in other plants, such as *Arabidopsis*, *Radish* and *Mikania micrantha* [7,9,10,14,15,16,17,18,19]. In addition, the other compounds included, *d*-pantothenic acid and hellicoside, were reported for the first time in plant biostimulants, which provided a reference for exploring new biostimulants and their effects. In summary, the above results might indicate that alkaloids, lipids, nucleotides and derivatives, phenolic acids, tannins and other compounds belonging to the biostimulants might act as positive regulators in the process of flower bud growth and development, just like the plant hormone biostimulants cytokinin, abscisic acid and indoleacetic acid [9,29,30].

## 4. Conclusions

A total of 204 biostimulant metabolites were identified in the flower buds of *Z. mioga* throughout the growth cycle. Based on the relative content information of the 204 identified compounds at each growth stage analysed by PCA, the four stages in the PCA plot were significantly different, and the contribution of the first two principal components PC1 and PC2 to the total variance was 71.02% and 8.31%, respectively, giving a total of 79.33%. The expression patterns of biostimulant-related metabolites in *Z. mioga* flower buds showed obvious phasic characteristics during the growth cycle. The identification of 122 differential metabolites and the visualization of their changing trends during the growth cycle were performed using OPLS-DA and K-means clustering analysis. The expression and accumulation of the top 50 differential compounds with the highest VIP value were clearly distinguished and significantly different between stages, and the mature stage was the highest point of abundance of almost all compounds. It was worth noting that the concentrations of these biostimulant-related compounds accumulated as the growth stage progressed, and the late developmental stage (SG3) was a key point for bud development. In addition, the correlation analysis results indicated that phenolic acids, nucleotides and derivatives, alkaloids, lipids and tannins had frequent and complex dialogues and synergistically participated in the growth and maturation of *Z. mioga* flower buds.

## Data Availability

The original contributions presented in this study can be found in the paper. For further information, please contact the corresponding author.

## Supplementary Materials

The following supporting information can be downloaded at https://www.mdpi.com/article/10.3390/metabo15060358/s1: Figure S1: The multimodal map of MRM metabolites in the flower buds of *Z. mioga*. A represents the positive ions; B indicates the negative ions. MRM, multiple reaction monitoring mode. Table S1: Primary metabolites related to biostimulants in the flower buds of *Z. mioga* at various developmental stages (SG1, SG2, SG3 and SG4 stage). Q1, quantitative ion; Q3, qualitative ion; and M, molecular weight. Relative content values are mean ± standard deviation for the three replicates. Following Duncan’s multiple comparisons, values with different superscript lowercase letters for the same metabolites of *Z. mioga* at different development stages indicate significant differences (*p* < 0.05). Table S2: Secondary metabolites related to biostimulants in the flower buds of *Z. mioga* at various developmental stages (SG1, SG2, SG3 and SG4 stage). Q1, quantitative ion; Q3, qualitative ion; and M, molecular weight. Relative content values are mean ± standard deviation for the three replicates. Following Duncan’s multiple comparisons, values with different superscript lowercase letters for the same metabolites of *Z. mioga* at different development stages indicate significant differences (*p* < 0.05). Figure S2: The hierarchical cluster analysis of the flower buds of *Z. mioga* by the stage of development. Figure S3: Krona plot of the classification and proportion of the top 50 differential metabolites with the highest VIP values. Nucleotides indicates the nucleotides and their derivatives. Table S3: The differential metabolites in the flower buds of *Z. mioga* in the stage of development obtained by the OPLS-DA method. OPLS-DA, orthogonal partial least squares discriminant analysis.
