# Supplementary material for: UPLC-ESI-QTRAP-MS-Based Metabolomics Revealed Changes in Biostimulant-Related Metabolite Profiles in Zingiber mioga Flower Buds During Development
Source: Metabolites. 2025 May 28;15(6):358. doi: 10.3390/metabo15060358 (PMC12195210; doi:10.3390/metabo15060358)
Supplement: Supplementary file 1 [file metabolites-15-00358-s001.zip › metabolites-3627540-supplementary.pdf]

metabolites

MDPI

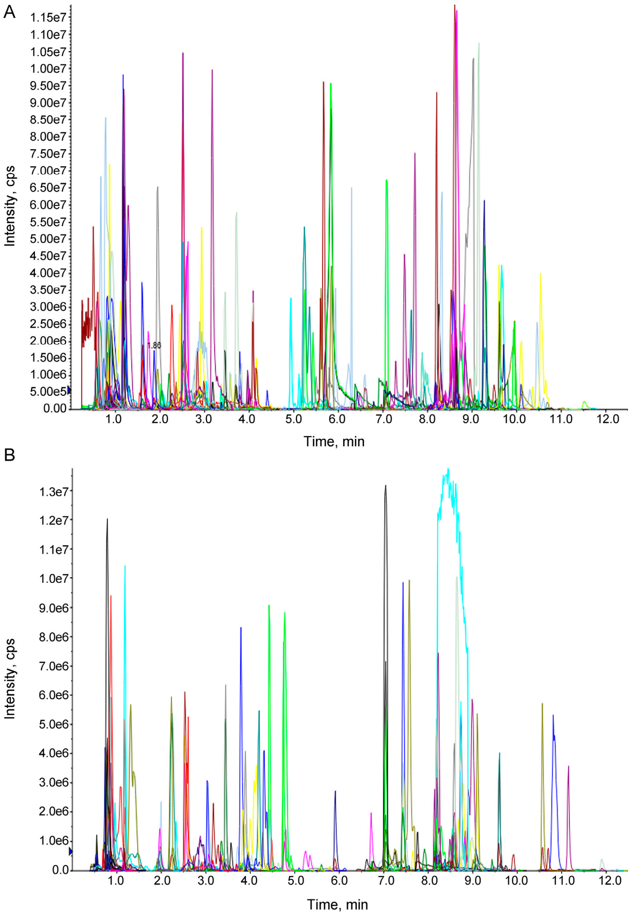

Figure S1: The multimodal map of MRM metabolites in the flower buds of *Z. mioga*. A represents the positive ions; B indicates the negative ions. MRM, multiple reaction monitoring mode.

Table S1: Primary metabolites related to biostimulants in the flower buds of *Z. mioga* at various developmental stages (SG1, SG2, SG3 and SG4 stage). Q1, quantitative ion; Q3, qualitative ion; and M, molecular weight. Relative content values are mean ± standard deviation for the three replicates. Following Duncan's multiple comparisons, values with different superscript lowercase letters for the same metabolites of *Z. mioga* at different development stages indicate significant differences ($P < 0.05$).

No.	Compounds	Identification of the components				Relative contents			
		Q1	Q3	M (Da)	Mode of ionization	GS1	GS2	GS3	GS4
Nucleotides and derivatives									
1	adenylthiomethylpentose	298.10	136.00	297.09	$[M+H]^+$	0.96±0.09^{a}	1.36±0.02^{b}	1.44±0.00^{b}	1.66±0.04^{c}
2	desoxyinosin-5'-phosphat	333.05	137.05	332.05	$[M+H]^+$	1.66±0.09^{a}	2.05±0.03^{b}	2.15±0.03^{b}	2.51±0.08^{c}

 https://doi.org/10.3390/metabo15060358

3	adenine	136.06	119.00	135.05	$[M+H]^+$	0.27±0.01^{a}	0.35±0.00^{b}	0.63±0.04^{c}	1.78±0.06^{d}
4	2'-deoxyguanosine	268.10	152.00	267.10	$[M+H]^+$	0.19±0.00^{a}	0.24±0.01^{a}	0.47±0.01^{b}	7.87±0.06^{c}
5	adenosine	268.10	136.06	267.10	$[M+H]^+$	0.02±0.01^{a}	0.05±0.01^{a}	0.32±0.03^{b}	1.10±0.04^{c}
6	6-methylmercaptopurine	167.10	121.10	166.03	$[M+H]^+$	0.38±0.02^{a}	0.45±0.01^{b}	0.47±0.01^{b}	0.79±0.02^{c}
7	5'-adenylic acid	348.07	136.00	347.06	$[M+H]^+$	0.48±0.02^{a}	0.65±0.02^{b}	0.78±0.01^{c}	0.98±0.03^{d}
8	Succinyladenosine	384.12	252.00	383.11	$[M+H]^+$	0.33±0.02^{c}	0.25±0.01^{b}	0.18±0.01^{a}	0.85±0.02^{d}
9	uridine diphosphoglucose	565.05	323.10	566.06	$[M-H]^-$	0.30±0.01^{b}	0.41±0.02^{c}	0.40±0.01^{c}	0.22±0.01^{a}
10	*N*, *N*-dimethylguanosine	312.13	180.00	311.12	$[M+H]^+$	0.22±0.01^{a}	0.23±0.02^{a}	0.33±0.01^{b}	0.66±0.01^{c}
11	5-aminoimidazole ribonucleotide	296.07	104.11	295.06	$[M+H]^+$	0.09±0.01^{a}	0.12±0.00^{b}	0.13±0.00^{c}	0.16±0.01^{d}
12	thymidine	243.10	127.00	242.09	$[M+H]^+$	0.02±0.00^{a}	0.03±0.00^{a}	0.10±0.01^{b}	1.88±0.03^{c}
13	cytidine	244.09	112.00	243.09	$[M+H]^+$	0.03±0.01^{a}	0.04±0.01^{a}	0.05±0.01^{a}	0.23±0.00^{b}
14	nicotinic acid adenine dinucleotide	664.12	136.20	663.11	$[M+H]^+$	0.09±0.01^{b}	0.09±0.00^{b}	0.11±0.01^{c}	0.06±0.00^{a}
15	guanosine 3',5'-cyclic monophosphate	344.04	150.00	345.05	$[M-H]^-$	0.00±0.00^{a}	0.01±0.01^{a}	0.02±0.00^{a}	0.30±0.03^{b}
16	cytarabine	244.09	112.05	243.09	$[M+H]^+$	0.03±0.01^{a}	0.03±0.01^{a}	0.05±0.00^{b}	0.16±0.01^{c}
17	adenosine 5'-diphosphate	426.00	159.00	427.03	$[M-H]^-$	0.08±0.01^{a}	0.12±0.00^{b}	0.14±0.01^{c}	0.11±0.01^{b}
18	2'-*O*-methyladenosine	282.12	136.06	281.11	$[M+H]^+$	0.04±0.00^{a}	0.04±0.00^{a}	0.06±0.01^{b}	0.18±0.01^{c}
19	2'-deoxyadenosine	252.11	136.00	251.10	$[M+H]^+$	0.00±0.00^{a}	0.01±0.01^{a}	0.03±0.01^{a}	0.78±0.10^{b}
20	2-deoxyribose-1-phosphate	213.10	97.00	214.02	$[M-H]^-$	0.02±0.01^{a}	0.03±0.01^{a}	0.04±0.01^{b}	0.11±0.01^{c}
21	isoguanine	152.06	135.00	151.05	$[M+H]^+$	0.02±0.01^{a}	0.03±0.00^{a}	0.03±0.00^{a}	0.08±0.01^{b}
22	2-aminopurine	136.06	118.90	135.05	$[M+H]^+$	0.00±0.00^{a}	0.01±0.00^{b}	0.04±0.01^{c}	0.31±0.01^{d}
23	uridine	243.06	110.00	244.07	$[M-H]^-$	0.01±0.00^{a}	0.01±0.00^{a}	0.05±0.01^{b}	0.16±0.01^{c}
24	guanine	152.06	135.00	151.05	$[M+H]^+$	0.02±0.01^{a}	0.02±0.00^{a}	0.04±0.01^{b}	0.11±0.01^{c}
25	uridine 5'-monophosphate	323.03	211.00	324.04	$[M-H]^-$	0.02±0.00^{a}	0.04±0.01^{b}	0.04±0.00^{b}	0.10±0.01^{c}
26	triphosphopyridine nucleotide	742.10	620.10	743.08	$[M-H]^-$	0.04±0.00^{c}	0.02±0.00^{b}	0.02±0.01^{b}	0.01±0.01^{a}
27	xanthosine	283.07	151.00	284.08	$[M-H]^-$	0.04±0.00^{a}	0.08±0.01^{b}	0.10±0.01^{c}	0.14±0.01^{d}
28	1-methyladenine	150.08	133.00	149.07	$[M+H]^+$	0.02±0.00^{a}	0.03±0.00^{a}	0.03±0.00^{a}	0.05±0.00^{b}
29	7-methylguanosine	298.10	166.30	297.11	$[M+H]^+$	0.04±0.01^{a}	0.04±0.01^{a}	0.04±0.01^{a}	0.08±0.00^{b}
30	cytosine	112.05	95.00	111.04	$[M+H]^+$	0.03±0.00^{c}	0.02±0.00^{b}	0.01±0.01^{a}	0.07±0.00^{d}
31	uridine 5'-diphosphate	402.99	159.00	404.00	$[M-H]^-$	0.03±0.00^{b}	0.04±0.01^{c}	0.04±0.00^{c}	0.02±0.00^{a}

32	deoxyadenylic acid	332.08	136.00	331.07	[M+H]$^{+}$	0.03±0.00^{a}	0.03±0.01^{a}	0.03±0.01^{a}	0.02±0.01^{a}
33	xanthine	151.03	151.00	152.03	[M-H]$^{-}$	0.03±0.00^{a}	0.07±0.01^{b}	0.17±0.01^{c}	0.21±0.01^{d}
34	uracil	111.02	80.00	112.03	[M-H]$^{-}$	0.02±0.00^{a}	0.02±0.01^{a}	0.02±0.00^{a}	0.03±0.00^{b}
35	arabinosylhypoxanthine	267.07	135.00	268.08	[M-H]$^{-}$	0.01±0.00^{a}	0.02±0.00^{a}	0.08±0.01^{b}	0.17±0.01^{c}
36	cytidylic acid	324.06	112.00	323.05	[M+H]$^{+}$	0.01±0.01^{a}	0.01±0.00^{a}	0.01±0.00^{a}	0.05±0.01^{b}
37	cordycepin DL-2-aminoadipic acid	252.11	69.00	251.10	[M+H]$^{+}$	0.00±0.00^{a}	0.00±0.00^{a}	0.01±0.00^{b}	0.26±0.01^{c}
38	guanosine 5'-monophosphate	364.07	152.10	363.06	[M+H]$^{+}$	0.01±0.01^{a}	0.02±0.00^{a}	0.03±0.00^{b}	0.05±0.01^{c}
39	2'-deoxycytidine	228.10	112.00	227.09	[M+H]$^{+}$	0.00±0.00^{a}	0.00±0.00^{a}	0.00±0.00^{a}	0.07±0.01^{b}
40	*β*-pseudouridine	243.06	153.00	244.07	[M-H]$^{-}$	0.01±0.00^{a}	0.01±0.00^{a}	0.01±0.00^{a}	0.03±0.00^{b}
41	5-methylcytosine	126.07	109.00	125.06	[M+H]$^{+}$	0.00±0.00^{a}	0.00±0.00^{a}	0.00±0.00^{a}	0.02±0.00^{b}
42	hypoxanthine	137.05	119.00	136.04	[M+H]$^{+}$	0.00±0.00^{a}	0.00±0.00^{a}	0.00±0.00^{a}	0.02±0.01^{b}
43	2'-deoxycytidine-5'-monophosphate	306.05	110.00	307.06	[M-H]$^{-}$	0.00±0.00^{a}	0.00±0.00^{a}	0.00±0.00^{a}	0.01±0.01^{b}
44	allopurinol	137.05	110.00	136.04	[M+H]$^{+}$	0.00±0.00^{a}	0.00±0.00^{a}	0.00±0.00^{a}	0.02±0.01^{b}
45	2'-deoxyuridine	227.10	184.00	228.08	[M-H]$^{-}$	0.00±0.00^{a}	0.00±0.00^{a}	0.00±0.00^{a}	0.05±0.01^{b}
46	uric acid	167.02	124.01	168.03	[M-H]$^{-}$	0.00±0.00^{a}	0.00±0.00^{a}	0.01±0.00^{b}	0.03±0.01^{c}
47	7-methylguanine	166.07	149.05	165.07	[M+H]$^{+}$	0.00±0.00^{a}	0.00±0.00^{a}	0.00±0.00^{a}	0.01±0.00^{b}
Lipids									
48	LysoPC 16:1	494.32	184.07	493.32	[M+H]$^{+}$	1.03±0.14^{a}	2.68±0.10^{b}	3.47±0.06^{c}	6.89±0.28^{d}
49	LysoPC 16:0	496.34	184.07	495.33	[M+H]$^{+}$	0.54±0.02^{a}	0.67±0.01^{b}	0.73±0.01^{c}	1.00±0.05^{d}
50	hexadecylsphingosine	274.27	256.27	273.27	[M+H]$^{+}$	0.48±0.01^{a}	0.55±0.01^{b}	0.60±0.01^{c}	0.67±0.01^{d}
51	LysoPC 18:1	522.36	184.07	521.35	[M+H]$^{+}$	0.35±0.01^{a}	0.45±0.02^{b}	1.02±0.02^{c}	1.18±0.02^{d}
52	LysoPC 18:0	524.37	184.07	523.36	[M+H]$^{+}$	0.15±0.01^{a}	0.31±0.01^{b}	0.37±0.01^{c}	0.59±0.01^{d}
53	LysoPC 18:2	520.34	184.07	519.33	[M+H]$^{+}$	0.22±0.01^{a}	0.34±0.02^{b}	0.53±0.01^{c}	0.71±0.01^{d}
54	LysoPE 18:2	478.29	337.27	477.29	[M+H]$^{+}$	0.20±0.00^{a}	0.68±0.05^{b}	1.00±0.08^{c}	1.94±0.09^{d}
55	LysoPC 15:1	480.31	184.07	479.30	[M+H]$^{+}$	0.14±0.00^{a}	0.29±0.02^{b}	0.67±0.07^{c}	1.19±0.06^{d}
56	LysoPE 16:0	454.29	313.27	453.29	[M+H]$^{+}$	0.09±0.01^{b}	0.05±0.00^{a}	0.06±0.00^{a}	0.15±0.00^{c}
57	LysoPC 17:1	508.34	184.07	507.33	[M+H]$^{+}$	0.10±0.02^{a}	0.21±0.01^{b}	0.41±0.02^{c}	0.69±0.02^{d}
58	LysoPC 18:3	518.32	184.07	517.32	[M+H]$^{+}$	0.09±0.00^{a}	0.19±0.00^{b}	0.31±0.01^{c}	0.43±0.01^{d}
59	LysoPC 16:2	492.31	184.07	491.30	[M+H]$^{+}$	0.09±0.00^{a}	0.23±0.00^{b}	0.44±0.04^{c}	0.67±0.04^{d}

60	LysoPE 18:3	476.28	335.26	475.27	$[M+H]^+$	0.06±0.00[a]	0.26±0.01[b]	0.37±0.01[c]	0.73±0.01[d]
61	LysoPC 20:2	548.37	184.07	547.36	$[M+H]^+$	0.06±0.00[a]	0.18±0.00[b]	0.24±0.01[c]	0.37±0.01[d]
62	LysoPC 17:2	506.32	184.07	505.32	$[M+H]^+$	0.06±0.00[a]	0.13±0.00[b]	0.24±0.01[c]	0.37±0.01[d]
63	LysoPC 14:0	468.31	184.07	467.30	$[M+H]^+$	0.05±0.00[a]	0.08±0.00[a]	0.15±0.00[b]	0.27±0.03[c]
64	LysoPG 16:0	483.27	255.23	484.28	$[M-H]^-$	0.02±0.00[a]	0.06±0.00[b]	0.07±0.00[c]	0.08±0.00[d]
65	1-linoleoylglycerol-2,3-di-*O*-glucoside	679.39	263.24	678.38	$[M+H]^+$	0.04±0.00[a]	0.10±0.00[b]	0.13±0.00[c]	0.19±0.00[d]
66	1-*O*-*p*-hydroxycinnamoyl-3-*O*-caffeoylglycerol	401.12	163.04	400.12	$[M+H]^+$	0.04±0.00[c]	0.03±0.00[b]	0.02±0.00[a]	0.02±0.00[a]
67	1-oleoyl-Sn-glycerol	357.30	265.40	356.29	$[M+H]^+$	0.03±0.01[a]	0.11±0.01[b]	0.15±0.01[c]	0.26±0.02[d]
68	2-linoleoylglycerol-1,3-di-*O*-glucoside	679.39	263.24	678.38	$[M+H]^+$	0.03±0.00[a]	0.09±0.00[b]	0.13±0.00[c]	0.20±0.00[d]
69	gingerglycolipid A	675.36	397.14	676.37	$[M-H]^-$	0.03±0.00[a]	0.17±0.01[b]	0.22±0.01[c]	0.32±0.01[d]
70	LysoPC 17:0	510.36	184.07	509.35	$[M+H]^+$	0.02±0.00[a]	0.03±0.00[b]	0.05±0.00[c]	0.12±0.01[d]
71	LysoPC 20:1	550.39	184.07	549.38	$[M+H]^+$	0.02±0.00[a]	0.02±0.00[a]	0.04±0.00[b]	0.07±0.00[c]
72	2-*α*-linolenoyl-glycerol	353.27	261.22	352.26	$[M+H]^+$	0.02±0.00[a]	0.03±0.00[b]	0.04±0.00[c]	0.11±0.01[d]
73	1-*O*-feruloyl-3-*O*-caffeoylglycerol	429.12	429.12	430.13	$[M-H]^-$	0.02±0.00[b]	0.01±0.00[a]	0.01±0.00[a]	0.01±0.00[a]
74	choline alfoscerate	258.11	104.11	257.10	$[M+H]^+$	0.01±0.00[a]	0.03±0.00[b]	0.03±0.00[b]	0.05±0.01[c]
75	1-*α*-linolenoyl-glycerol	353.27	261.22	352.26	$[M+H]^+$	0.01±0.00[a]	0.01±0.00[a]	0.02±0.00[b]	0.03±0.00[c]
76	LysoPC 15:0	482.32	184.07	481.32	$[M+H]^+$	0.02±0.00[a]	0.02±0.00[a]	0.05±0.00[b]	0.11±0.00[c]
77	2-linoleoylglycerol	355.28	263.24	354.28	$[M+H]^+$	0.01±0.00[a]	0.01±0.00[a]	0.02±0.00[a]	0.07±0.00[b]
78	LysoPC 19:1	536.37	184.07	535.36	$[M+H]^+$	0.01±0.00[a]	0.01±0.00[a]	0.03±0.00[b]	0.05±0.00[c]
79	2-*α*-linolenoyl-glycerol-1,3-di-*O*-glucoside	677.37	261.22	676.37	$[M+H]^+$	0.01±0.00[a]	0.07±0.00[b]	0.09±0.01[c]	0.15±0.01[d]
80	1-*α*-linolenoyl-glycerol-2,3-di-*O*-glucoside	677.37	261.22	676.37	$[M+H]^+$	0.01±0.00[a]	0.06±0.00[b]	0.08±0.01[c]	0.12±0.01[d]
81	1-linoleoylglycerol	355.28	263.24	354.28	$[M+H]^+$	0.00±0.00[a]	0.00±0.00[a]	0.01±0.00[b]	0.02±0.00[c]
82	LysoPC 18:4	516.30	184.07	515.30	$[M+H]^+$	0.01±0.00[a]	0.03±0.00[b]	0.06±0.00[c]	0.10±0.00[d]
83	LysoPC 19:2	534.36	184.07	533.35	$[M+H]^+$	0.01±0.00[a]	0.02±0.00[b]	0.03±0.00[c]	0.05±0.00[d]
84	LysoPE 18:1	480.31	339.29	479.30	$[M+H]^+$	0.01±0.00[a]	0.02±0.00[b]	0.02±0.00[b]	0.04±0.00[c]
85	2-*α*-linolenoyl-glycerol-1-*O*-glucoside	515.32	261.22	514.31	$[M+H]^+$	0.01±0.00[a]	0.04±0.00[b]	0.05±0.00[c]	0.08±0.01[d]
86	LysoPC 20:3	546.36	184.07	545.35	$[M+H]^+$	0.00±0.00[a]	0.01±0.00[b]	0.02±0.00[c]	0.03±0.00[d]
87	LysoPE 16:1	452.28	311.26	451.27	$[M+H]^+$	0.00±0.00[a]	0.01±0.00[b]	0.01±0.00[b]	0.12±0.01[c]
88	LysoPE 16:0	454.29	313.27	453.29	$[M+H]^+$	0.00±0.00[a]	0.00±0.00[a]	0.00±0.00[a]	0.02±0.00[b]

89	LysoPE 17:1	466.29	325.27	465.29	$[M+H]^+$	$0.00±0.00^{a}$	$0.00±0.00^{a}$	$0.00±0.00^{a}$	$0.02±0.00^{b}$
90	2-linoleoylglycerol-1-*O*-glucoside	517.34	263.24	516.33	$[M+H]^+$	$0.00±0.00^{a}$	$0.01±0.00^{b}$	$0.01±0.00^{b}$	$0.01±0.00^{b}$
Others									
91	*d*-pantothenic acid	220.12	202.10	219.11	$[M+H]^+$	$1.11±0.01^{d}$	$0.97±0.00^{c}$	$0.91±0.01^{b}$	$0.76±0.01^{a}$
92	nicotinic acid riboside	256.20	124.10	256.08	$[M+H]^+$	$0.17±0.01^{a}$	$0.20±0.01^{b}$	$0.22±0.01^{b}$	$0.33±0.01^{c}$
93	nicotinamide	123.06	80.05	122.05	$[M+H]^+$	$0.13±0.01^{a}$	$0.16±0.00^{b}$	$0.17±0.01^{c}$	$0.29±0.01^{d}$
94	pyridoxine	170.08	134.10	169.07	$[M+H]^+$	$0.02±0.00^{a}$	$0.07±0.01^{b}$	$0.08±0.01^{c}$	$0.21±0.01^{d}$
95	4-pyridoxic acid-*O*-glucoside	346.11	184.10	345.11	$[M+H]^+$	$0.07±0.00^{b}$	$0.08±0.00^{b}$	$0.08±0.01^{b}$	$0.06±0.01^{a}$
96	pyridoxine-5'-*O*-glucoside	332.13	152.07	331.13	$[M+H]^+$	$0.07±0.01^{b}$	$0.07±0.01^{b}$	$0.06±0.01^{b}$	$0.04±0.01^{a}$
97	vitamin B_3	124.04	78.00	123.03	$[M+H]^+$	$0.02±0.00^{a}$	$0.02±0.00^{a}$	$0.02±0.00^{a}$	$0.05±0.01^{b}$
98	4-pyridoxic acid	184.06	148.00	183.05	$[M+H]^+$	$0.01±0.00^{a}$	$0.01±0.00^{a}$	$0.01±0.00^{a}$	$0.02±0.00^{b}$

Table S2: Secondary metabolites related to biostimulants in the flower buds of *Z. mioga* at various developmental stages (SG1, SG2, SG3 and SG4 stage). Q1, quantitative ion; Q3, qualitative ion; and M, molecular weight. Relative content values are mean ± standard deviation for the three replicates. Following Duncan's multiple comparisons, values with different superscript lowercase letters for the same metabolites of *Z. mioga* at different developmental stages indicate significant differences ($P < 0.05$).

No.	Compounds	Identification of the components				Relative contents			
		Q1	Q3	M (Da)	Mode of ionization	GS1	GS2	GS3	GS4
Phenolic acids									
1	sinapoylglucuronic acid	399.09	205.04	400.10	$[M-H]^-$	$0.56±0.02^{a}$	$1.43±0.02^{b}$	$1.60±0.03^{c}$	$2.53±0.04^{d}$
2	3,4,5-trimethoxyphenyl-1-*O*-glucoside	329.12	167.00	330.13	$[M-H]^-$	$0.32±0.00^{a}$	$0.36±0.01^{b}$	$0.37±0.01^{b}$	$0.41±0.01^{c}$
3	trihydroxycinnamoylquinic acid	371.10	249.06	372.11	$[M-H]^-$	$0.26±0.00^{a}$	$0.26±0.01^{a}$	$0.30±0.00^{b}$	$0.35±0.01^{c}$
4	bis-(2-ethylhexyl)-phthalate	391.28	149.02	390.28	$[M+H]^+$	$0.12±0.00^{a}$	$0.13±0.00^{b}$	$0.13±0.00^{b}$	$0.16±0.00^{c}$
5	vanillin	151.04	136.00	152.05	$[M-H]^-$	$0.15±0.00^{a}$	$0.20±0.01^{b}$	$0.22±0.01^{c}$	$0.14±0.00^{a}$
6	syringic acid	197.05	123.00	198.05	$[M-H]^-$	$0.05±0.00^{a}$	$0.05±0.00^{a}$	$0.14±0.00^{b}$	$0.17±0.00^{c}$
7	diisooctyl phthalate	391.28	149.02	390.28	$[M+H]^+$	$0.09±0.00^{a}$	$0.11±0.00^{b}$	$0.12±0.00^{c}$	$0.15±0.00^{d}$
8	anthranilate-1-*O*-sophoroside	460.10	117.90	461.15	$[M-H]^-$	$0.13±0.00^{d}$	$0.04±0.00^{b}$	$0.02±0.00^{a}$	$0.08±0.00^{c}$
9	*β*-hydroxypropiosyringone	227.09	181.09	226.08	$[M+H]^+$	$0.11±0.00^{c}$	$0.04±0.00^{a}$	$0.07±0.00^{b}$	$0.14±0.00^{d}$
10	protocatechualdehyde	137.02	93.04	138.03	$[M-H]^-$	$0.05±0.00^{a}$	$0.06±0.00^{b}$	$0.13±0.00^{c}$	$0.24±0.01^{d}$
11	chlorogenic acid	353.09	191.01	354.10	$[M-H]^-$	$0.09±0.00^{c}$	$0.07±0.00^{b}$	$0.04±0.00^{a}$	$0.03±0.00^{a}$
12	4-hydroxybenzoic acid	137.02	93.00	138.03	$[M-H]^-$	$0.04±0.00^{a}$	$0.06±0.00^{b}$	$0.11±0.00^{c}$	$0.21±0.00^{d}$
13	vanillic acid	167.03	108.02	168.04	$[M-H]^-$	$0.05±0.00^{a}$	$0.05±0.00^{a}$	$0.11±0.01^{b}$	$0.20±0.01^{c}$

14	3,4'-dihydroxy-3',5'-dimethoxypropiophenone	227.09	181.05	226.08	[M+H]$^{+}$	0.09±0.01^{b}	0.08±0.01^{b}	0.04±0.00^{a}	0.04±0.00^{a}
15	terephthalic acid	165.02	121.03	166.03	[M-H]$^{-}$	0.08±0.00^{c}	0.04±0.00^{a}	0.05±0.00^{b}	0.05±0.00^{b}
16	sinapaldehyde	207.07	192.00	208.07	[M-H]$^{-}$	0.03±0.00^{a}	0.07±0.00^{b}	0.09±0.00^{c}	0.03±0.00^{a}
17	vanillic acid-4-*O*-glucoside	329.09	209.04	330.10	[M-H]$^{-}$	0.07±0.01^{d}	0.05±0.00^{c}	0.04±0.00^{b}	0.03±0.00^{a}
18	2-formamidobenzoate	164.04	120.00	165.04	[M-H]$^{-}$	0.04±0.00^{a}	0.04±0.00^{a}	0.07±0.00^{b}	0.07±0.00^{b}
19	centrolobol	301.18	131.05	300.17	[M+H]$^{+}$	0.04±0.00^{a}	0.05±0.00^{b}	0.05±0.00^{b}	0.06±0.00^{c}
20	cryptochlorogenic acid	353.09	191.05	354.10	[M-H]$^{-}$	0.06±0.00^{b}	0.06±0.00^{b}	0.02±0.00^{a}	0.02±0.00^{a}
21	*p*-coumaric acid	165.05	119.00	164.05	[M+H]$^{+}$	0.05±0.00^{d}	0.02±0.00^{a}	0.03±0.00^{b}	0.04±0.00^{c}
22	4-*O*-glucosyl-4-hydroxybenzoic acid	299.08	137.02	300.08	[M-H]$^{-}$	0.04±0.00^{b}	0.05±0.00^{c}	0.05±0.00^{c}	0.02±0.00^{a}
23	1-*O*-salicyl-*d*-glucose	299.08	137.02	300.08	[M-H]$^{-}$	0.02±0.00^{a}	0.05±0.00^{b}	0.05±0.00^{b}	0.02±0.00^{a}
24	ferulic acid	193.05	134.01	194.06	[M-H]$^{-}$	0.03±0.00^{a}	0.04±0.00^{a}	0.05±0.00^{b}	0.12±0.01^{c}
25	vanillylmandelic acid	197.00	153.00	198.05	[M-H]$^{-}$	0.03±0.00^{b}	0.02±0.00^{a}	0.02±0.00^{a}	0.03±0.00^{b}
26	1-*O*-glucosyl sinapate	385.11	205.00	386.12	[M-H]$^{-}$	0.04±0.00^{d}	0.03±0.00^{c}	0.02±0.00^{b}	0.01±0.00^{a}
27	4-methoxycinnamaldehyde	163.08	91.10	162.07	[M+H]$^{+}$	0.03±0.00^{a}	0.03±0.00^{a}	0.03±0.00^{a}	0.03±0.00^{a}
28	4-*O*-glucosyl-3,4-dihydroxybenzyl alcohol	301.09	139.05	302.10	[M-H]$^{-}$	0.02±0.00^{a}	0.03±0.00^{b}	0.03±0.00^{b}	0.03±0.00^{b}
29	koaburaside	331.10	153.02	332.11	[M-H]$^{-}$	0.02±0.00^{b}	0.03±0.00^{c}	0.01±0.00^{a}	0.01±0.00^{a}
30	salicylic acid-2-*O*-glucoside	299.10	137.10	300.09	[M-H]$^{-}$	0.02±0.00^{a}	0.02±0.00^{a}	0.02±0.00^{a}	0.02±0.00^{a}
31	2,6-di-tert-butylphenol	205.16	189.13	206.17	[M-H]$^{-}$	0.02±0.00^{a}	0.03±0.00^{b}	0.04±0.00^{c}	0.03±0.00^{b}
32	[10]-paradol	335.26	151.07	334.25	[M+H]$^{+}$	0.02±0.00^{b}	0.02±0.00^{b}	0.01±0.00^{a}	0.01±0.00^{a}
33	caffeic acid	179.03	135.05	180.04	[M-H]$^{-}$	0.02±0.00^{b}	0.01±0.00^{a}	0.01±0.00^{a}	0.01±0.00^{a}
34	1-*O*-gentisoyl-*d*-glucoside	315.07	153.00	316.08	[M-H]$^{-}$	0.02±0.00^{a}	0.04±0.01^{b}	0.04±0.00^{b}	0.03±0.00 ^{b}
35	coniferin	341.12	179.00	342.13	[M-H]$^{-}$	0.02±0.00^{b}	0.01±0.00^{a}	0.01±0.00^{a}	0.01±0.00^{a}
36	protocatechuic acid-4-*O*-glucoside	315.07	153.02	316.08	[M-H]$^{-}$	0.01±0.00^{a}	0.03±0.00^{b}	0.03±0.00^{b}	0.03±0.00^{b}
37	2,4-dihydroxybenzoic acid	153.02	109.03	154.03	[M-H]$^{-}$	0.01±0.00^{a}	0.02±0.00^{b}	0.07±0.00^{c}	0.16±0.00^{d}
38	2-*O*-salicyl-6-*O*-galloyl-*d*-glucose	451.09	313.05	452.10	[M-H]$^{-}$	0.01±0.00^{a}	0.02±0.00^{b}	0.02±0.00^{b}	0.02±0.00^{b}
39	2-feruloyl-sn-glycerol	269.10	177.05	268.10	[M+H]$^{+}$	0.01±0.00^{a}	0.02±0.00^{b}	0.02±0.00^{b}	0.01±0.00^{a}
40	phthalic anhydride	149.02	65.04	148.02	[M+H]$^{+}$	0.01±0.00^{a}	0.02±0.00^{b}	0.02±0.00^{b}	0.02±0.00^{b}
41	1'-*O*-(3,4-dihydroxyphenethyl)-*O*-caffeoyl-glucoside	477.14	179.03	478.15	[M-H]$^{-}$	0.01±0.00^{a}	0.01±0.00^{a}	0.18±0.01^{b}	0.26±0.01^{c}
42	protocatechuic acid	153.02	109.03	154.03	[M-H]$^{-}$	0.01±0.00^{a}	0.02±0.00^{b}	0.05±0.00^{c}	0.14±0.01^{d}
43	Di-O-glucosylquinic acid	515.16	341.11	516.17	[M-H]$^{-}$	0.01±0.00^{a}	0.01±0.00^{a}	0.01±0.00^{a}	0.01±0.00^{a}
44	syringaldehyde	181.05	151.00	182.06	[M-H]$^{-}$	0.01±0.00^{a}	0.01±0.00^{a}	0.01±0.00^{a}	0.01±0.00^{a}
45	6-*O*-caffeoylarbutin	433.11	271.06	434.12	[M-H]$^{-}$	0.01±0.00^{a}	0.02±0.00^{b}	0.03±0.00^{c}	0.02±0.00^{b}
46	1-*O*-feruloyl-*d*-glucose	355.10	193.05	356.11	[M-H]$^{-}$	0.01±0.00^{a}	0.01±0.00^{a}	0.01±0.00^{a}	0.01±0.00^{a}
47	gentisic acid	153.02	109.03	154.03	[M-H]$^{-}$	0.01±0.00^{a}	0.01±0.00^{a}	0.03±0.00^{b}	0.08±0.00^{c}
48	salicylic acid	137.03	108.02	138.03	[M-H]$^{-}$	0.00±0.00^{a}	0.01±0.00^{b}	0.01±0.00^{b}	0.02±0.00^{c}
49	brevifolin-carboxylic acid	291.01	247.00	292.02	[M-H]$^{-}$	0.00±0.00^{a}	0.01±0.00^{b}	0.02±0.00^{c}	0.01±0.00^{b}
50	5-*O*-feruloylquinic acid	735.22	367.11	368.11	[2M-H]$^{-}$	0.00±0.00^{a}	0.00±0.00^{a}	0.00±0.00^{a}	0.01±0.00^{b}
51	benzamide	122.00	105.00	121.05	[M+H]$^{+}$	0.00±0.00^{a}	0.00±0.00^{a}	0.00±0.00^{a}	0.01±0.00^{b}
52	[4]-gingerdiol	353.23	151.07	352.19	[M+H]$^{+}$	0.00±0.00^{a}	0.02±0.00^{b}	0.02±0.00^{b}	0.03±0.00^{c}
53	phloretic acid	165.06	119.05	166.06	[M-H]$^{-}$	0.00±0.00^{a}	0.00±0.00^{a}	0.00±0.00^{a}	0.01±0.00^{b}
Alkaloids									
54	choline	104.10	60.10	103.09	[M+H]$^{+}$	3.16±0.02^{a}	3.42±0.04 ^{b}	4.08±0.07^{c}	6.41±0.23^{d}
55	*n*-benzylmethylene isomethylamine	120.08	103.05	119.07	[M+H]$^{+}$	2.32±0.06^{a}	2.60±0.02^{b}	2.77±0.07^{c}	4.18±0.10^{d}

56	6-deoxyfagomine	132.10	57.10	131.10	[M+H]$^{+}$	1.76±0.02^{a}	1.92±0.06^{b}	2.05±0.03^{c}	2.51±0.03^{d}
57	piperidine	86.10	69.20	85.09	[M+H]+	1.55±0.04^{a}	1.65±0.03^{b}	1.85±0.05^{c}	2.10±0.04^{d}
58	spermine	203.22	83.00	202.22	[M+H]+	1.48±0.08^{d}	1.11±0.01^{c}	0.98±0.01^{b}	0.48±0.03^{a}
59	4-hydroxymandelonitrile	150.06	61.01	149.05	[M+H]+	0.98±0.02^{a}	1.08±0.04^{b}	1.26±0.01^{c}	1.70±0.02^{d}
60	histidinol	142.10	124.09	141.09	[M+H]$^{+}$	0.62±0.02^{d}	0.56±0.01^{c}	0.49±0.01^{b}	0.24±0.00^{a}
61	trigonelline	138.05	94.07	137.05	[M+H]$^{+}$	0.58±0.02^{b}	0.57±0.01^{b}	0.58±0.01^{b}	0.43±0.01^{a}
62	1-methoxy-indole-3-acetamide	205.10	146.06	204.09	[M+H]$^{+}$	0.21±0.01^{a}	0.24±0.01^{b}	0.33±0.01^{c}	0.40±0.01^{d}
63	3-hydroxypropyl palmitate glc-glucosamine	672.42	331.29	671.41	[M+H]$^{+}$	0.06±0.00^{a}	0.08±0.00^{b}	0.14±0.00^{c}	0.15±0.00^{d}
64	2-aminoadipicacid	162.08	98.00	161.07	[M+H]$^{+}$	0.11±0.01^{a}	0.15±0.01^{b}	0.22±0.01^{c}	0.38±0.02^{d}
65	4,5,6-Trihydroxy-2-cyclohexen-1-ylideneacetonitrile	168.07	105.03	167.06	[M+H]$^{+}$	0.09±0.01^{a}	0.10±0.00^{a}	0.11±0.01^{b}	0.20±0.01^{c}
66	methoxyindoleacetic acid	206.10	147.50	205.07	[M+H]$^{+}$	0.05±0.00^{a}	0.06±0.00^{b}	0.09±0.01^{c}	0.11±0.01^{d}
67	betaine	118.09	59.00	117.08	[M+H]$^{+}$	0.07±0.00^{a}	0.07±0.00^{b}	0.09±0.00^{c}	0.11±0.00^{d}
68	putrescine	89.11	72.08	88.10	[M+H]$^{+}$	0.03±0.00^{a}	0.03±0.01^{a}	0.05±0.01^{b}	0.10±0.00^{c}
69	uridine 5'-diphospho-*n*-acetylglucosamine	606.08	384.98	607.08	[M-H]$^{-}$	0.02±0.00^{a}	0.02±0.00^{a}	0.03±0.00^{b}	0.02±0.00^{a}
70	10-formyltetrahydrofuran	474.17	327.00	473.17	[M+H]$^{+}$	0.02±0.00^{b}	0.02±0.00^{b}	0.01±0.00^{a}	0.01±0.00^{a}
71	*O*-phosphocholine	184.00	125.00	184.07	[M]$^{+}$	0.01±0.01^{a}	0.07±0.01^{b}	0.10±0.01^{c}	0.09±0.01^{c}
72	*N′*, *N′′*, *N′′′*-p-coumaroyl-cinnamoyl-caffeoyl spermidine	584.28	325.00	583.27	[M+H]$^{+}$	0.00±0.00^{a}	0.00±0.00^{a}	0.02±0.00^{b}	0.04±0.00^{c}
73	indole	118.07	91.00	117.06	[M+H]$_{+}$	0.02±0.00^{a}	0.02±0.00^{a}	0.02±0.01^{a}	0.03±0.00^{b}
74	*O*-phosphorylethanolamine	140.01	78.96	141.02	[M-H]$_{-}$	0.01±0.01^{a}	0.02±0.01^{a}	0.03±0.00^{b}	0.02±0.00^{a}
75	agmatine	131.13	114.10	130.12	[M+H]$^{+}$	0.01±0.00^{a}	0.02±0.00^{b}	0.02±0.01^{b}	0.04±0.00^{c}
76	*N*-acetylputrescine	131.12	114.09	130.11	[M+H]$^{+}$	0.01±0.00^{a}	0.02±0.00^{b}	0.02±0.00^{b}	0.04±0.00^{c}
77	acetylcholine	147.13	87.20	146.12	[M+H]$^{+}$	0.01±0.01^{a}	0.02±0.01^{a}	0.02±0.01^{a}	0.02±0.00^{a}
78	phenethylamine	122.10	105.00	121.09	[M+H]$^{+}$	0.01±0.00^{a}	0.01±0.00^{a}	0.02±0.00^{b}	0.03±0.00^{c}
79	indole-3-carboxylic acid	160.04	116.00	161.05	[M-H]$^{-}$	0.00±0.00^{a}	0.00±0.00^{a}	0.01±0.00^{b}	0.03±0.01^{c}
80	*N*-oleoylethanolamine	326.31	62.06	325.30	[M+H]$^{+}$	0.00±0.00^{a}	0.00±0.00^{a}	0.00±0.00^{a}	0.03±0.00^{b}
81	indole-3-acetic acid	176.10	130.10	175.06	[M+H]$^{+}$	0.00±0.00^{a}	0.00±0.00^{a}	0.00±0.00^{a}	0.01±0.00^{b}
Tannins									
82	epitheaflavic acid-3-*O*-gallate	579.08	289.07	580.09	[M-H]$^{-}$	0.07±0.01^{a}	0.34±0.00^{b}	0.40±0.00^{c}	0.59±0.01^{d}
83	1-*O*-galloyl-*d*-glucose	331.07	169.01	332.07	[M-H]$^{-}$	0.02±0.00^{a}	0.02±0.00^{a}	0.02±0.00^{a}	0.02±0.00^{a}
Terpenoids									
84	pinusolidic acid	333.21	287.20	332.20	[M+H]$^{+}$	0.09±0.00^{a}	0.10±0.00^{b}	0.11±0.01^{b}	0.14±0.01^{c}
85	cafestol	317.21	299.00	316.20	[M+H]$^{+}$	0.12±0.01^{c}	0.12±0.01^{c}	0.10±0.00^{b}	0.07±0.01^{a}
86	galanal A	319.23	301.20	318.22	[M+H]$^{+}$	0.04±0.00^{a}	0.05±0.00^{b}	0.05±0.00^{b}	0.05±0.00^{b}
87	oxyphyllol D	251.17	191.14	252.17	[M-H]$^{-}$	0.03±0.00^{b}	0.02±0.00^{a}	0.02±0.00^{a}	0.02±0.00^{a}
88	2α-hydroxyursolic acid	471.35	471.35	472.36	[M-H]$^{-}$	0.00±0.00^{a}	0.00±0.00^{a}	0.02±0.00^{b}	0.03±0.00^{b}
89	camaldulenic acid	469.33	469.33	470.34	[M-H]$^{-}$	0.01±0.01^{a}	0.02±0.01^{b}	0.03±0.00^{b}	0.03±0.01^{b}
90	miogadial	317.21	287.20	318.22	[M-H]$^{-}$	0.01±0.00^{a}	0.01±0.00^{a}	0.03±0.01^{b}	0.02±0.01^{b}
**Others**									
91	scopoletin	193.05	133.00	192.04	[M+H]$^{+}$	0.01±0.00^{a}	0.01±0.00^{a}	0.01±0.00^{a}	0.01±0.00^{a}
92	2,3,5,4'-tetrahydroxystilbene-2-*O*-glucoside	405.12	243.10	406.13	[M-H]$^{-}$	0.01±0.00^{a}	0.02±0.00^{a}	0.03±0.00^{b}	0.03±0.01^{b}
93	2,4,6,4'-tetrahydroxy-stilbene-2-*O*-glucoside	405.12	243.10	406.13	[M-H]$^{-}$	0.01±0.00^{a}	0.02±0.00^{a}	0.03±0.01^{b}	0.02±0.00^{b}
94	dihydrocurcumin	371.15	177.05	370.14	[M+H]$^{+}$	0.15±0.01^{a}	0.17±0.01^{b}	0.18±0.01^{b}	0.22±0.01^{c}
95	sodium valproate	143.11	143.11	166.10	[M-Na]$^{-}$	0.09±0.01^{a}	0.10±0.01^{b}	0.11±0.00^{b}	0.12±0.00^{c}

96	dihydrodemethoxy curcumin	341.14	177.05	340.13	$[M+H]^+$	0.05±0.01^{c}	0.04±0.00^{b}	0.04±0.00^{b}	0.03±0.01^{a}
97	propyl 2-(trimethylammonio) ethyl phosphate	544.34	485.27	543.33	$[M+H]^+$	0.04±0.01^{a}	0.05±0.01^{a}	0.12±0.01^{b}	0.13±0.00^{c}
98	dihydrodidemethoxy curcumin	311.13	107.04	310.12	$[M+H]^+$	0.04±0.01^{a}	0.03±0.01^{a}	0.09±0.01^{b}	0.04±0.00^{a}
99	ligraminol B	597.22	387.14	598.23	$[M-H]^-$	0.03±0.01^{c}	0.02±0.00^{b}	0.01±0.00^{a}	0.01±0.00^{a}
100	eucommiol	187.10	169.09	188.11	$[M-H]^-$	0.01±0.00^{a}	0.02±0.00^{a}	0.04±0.00^{b}	0.13±0.01^{c}
101	vanilloloside	315.11	153.02	316.12	$[M-H]^-$	0.01±0.00^{a}	0.02±0.00^{b}	0.03±0.01^{b}	0.02±0.00^{b}
102	gingerenone A	357.17	177.09	356.16	$[M+H]^+$	0.01±0.00^{a}	0.01±0.00^{a}	0.02±0.00^{b}	0.01±0.01^{a}
103	officinarumane C	401.28	105.07	400.28	$[M+H]^+$	0.01±0.00^{a}	0.01±0.00^{a}	0.01±0.00^{a}	0.01±0.00^{a}
104	hellicoside	655.19	637.17	656.20	$[M-H]^-$	0.00±0.01^{a}	0.04±0.01^{b}	0.11±0.01^{c}	0.16±0.00^{d}
105	creatine	130.09	88.04	131.07	$[M-H]^-$	0.00±0.00^{a}	0.00±0.00^{a}	0.01±0.00^{b}	0.01±0.00^{b}
106	5-hydroxy-7-(4-hydroxy-3,5-dimethoxyphenyl)-1-(4-hydroxy-3-methoxyphenyl) heptan-3-one	403.17	179.07	404.18	$[M-H]^-$	0.00±0.00^{a}	0.01±0.01^{b}	0.02±0.01^{b}	0.02±0.00^{b}

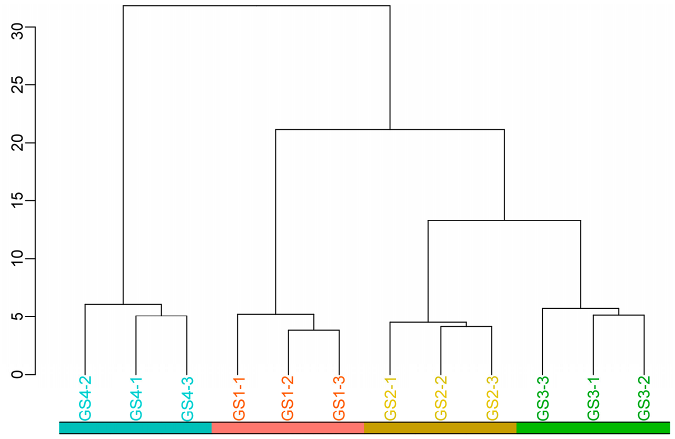

Figure S2: The hierarchical cluster analysis of the flower buds of *Z. mioga* by the stage of development.

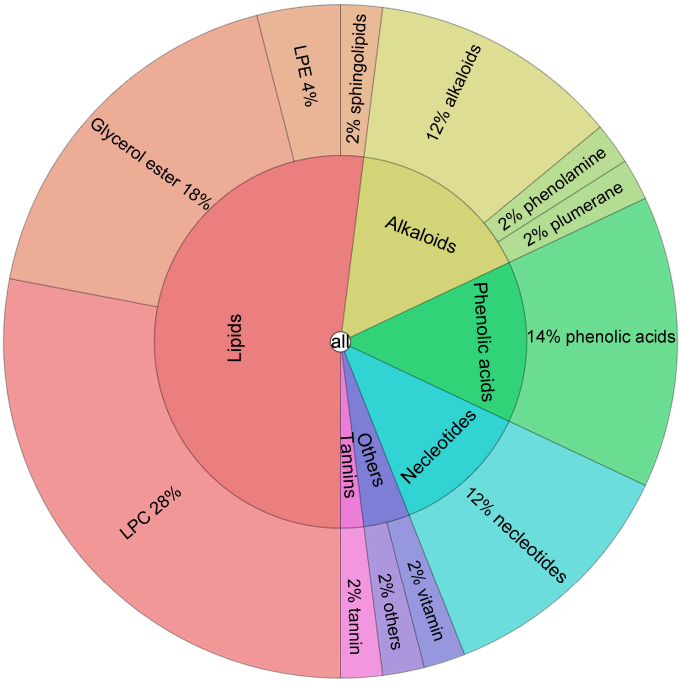

Figure S3: Krona plot of the classification and proportion of the top 50 differential metabolites with the highest VIP values. Nucleotides indicates the nucleotides and their derivatives.

Table S3: The differential metabolites in the flower buds of *Z. mioga* in the stage of development obtained by the OPLS-DA method. OPLS-DA, orthogonal partial least squares discriminant analysis.

No.	Formula	Compounds	VIP	*P*-value
1	$C_{26}H_{48}NO_7P$	LysoPC 18:3	1.215	0.000
2	$C_{33}H_{58}O_{14}$	2-linoleoylglycerol-1,3-di-*O*-glucoside	1.213	0.000
3	$C_{33}H_{58}O_{14}$	1-linoleoylglycerol-2,3-di-*O*-glucoside	1.211	0.000
4	$C_{10}H_{14}N_5O_7P$	5'-adenylic acid	1.211	0.000
5	$C_{28}H_{54}NO_7P$	LysoPC 20:2	1.209	0.000
6	$C_{26}H_{50}NO_7P$	LysoPC 18:2	1.208	0.000
7	$C_{25}H_{48}NO_7P$	LysoPC 17:2	1.206	0.000
8	$C_{16}H_{35}NO_2$	hexadecylsphingosine	1.206	0.000
9	$C_9H_{17}NO_5$	*d*-pantothenic acid	1.205	0.000
10	$C_{10}H_{12}N_4O_6$	xanthosine	1.204	0.000
11	$C_{33}H_{56}O_{14}$	2-α-linolenoyl-glycerol-1,3-di-*O*-glucoside	1.203	0.000
12	$C_{24}H_{46}NO_7P$	LysoPC 16:2	1.203	0.000
13	$C_{29}H_{36}O_{17}$	hellicoside	1.201	0.000
14	$C_{27}H_{52}NO_7P$	LysoPC 19:2	1.200	0.000
15	$C_{33}H_{56}O_{14}$	gingerglycolipid A	1.200	0.000
16	$C_{28}H_{52}NO_7P$	LysoPC 20:3	1.197	0.000

17	$C_5H_4N_4O_2$	xanthine	1.196	0.000
18	$C_{21}H_{20}O_8$	1-*O*-p-hydroxycinnamoyl-3-*O*-caffeoylglycerol	1.195	0.000
19	$C_{26}H_{54}NO_7P$	LysoPC 18:0	1.194	0.000
20	$C_8H_{11}N$	phenethylamine	1.192	0.000
21	$C_{11}H_{12}N_2O_2$	1-methoxy-indole-3-acetamide	1.191	0.000
22	$C_{21}H_{40}O_4$	1-oleoyl-Sn-glycerol	1.190	0.000
23	$C_{25}H_{50}NO_7P$	LysoPC 17:1	1.189	0.000
24	$C_{26}H_{46}NO_7P$	LysoPC 18:4	1.189	0.000
25	$C_{17}H_{20}O_{11}$	sinapoylglucuronic acid	1.189	0.000
26	$C_{10}H_{26}N_4$	spermine	1.188	0.000
27	$C_{33}H_{56}O_{14}$	1-α-linolenoyl-glycerol-2,3-di-*O*-glucoside	1.187	0.000
28	$C_{28}H_{20}O_{14}$	epitheaflavic acid-3-*O*-gallate	1.187	0.000
29	$C_{27}H_{46}O_9$	2-α-linolenoyl-glycerol-1-*O*-glucoside	1.186	0.000
30	$C_{23}H_{42}NO_7P$	LysoPE 18:3	1.185	0.000
31	$C_{11}H_{11}NO_3$	methoxyindoleacetic acid	1.184	0.000
32	$C_{23}H_{44}NO_7P$	LysoPE 18:2	1.183	0.000
33	$C_{10}H_{13}N_4O_7P$	desoxyinosin-5'-phosphat	1.182	0.000
34	$C_5H_{11}N$	piperidine	1.182	0.000
35	$C_{23}H_{46}NO_7P$	LysoPC 15:1	1.176	0.000
36	$C_{24}H_{48}NO_7P$	LysoPC 16:1	1.171	0.000
37	$C_5H_{11}NO_2$	betaine	1.170	0.000
38	$C_8H_{14}N_3O_7P$	5-aminoimidazole ribonucleotide	1.169	0.000
39	$C_{24}H_{38}O_4$	diisooctyl Phthalate	1.168	0.000
40	$C_{14}H_{18}O_9$	vanillic acid-4-*O*-glucoside	1.168	0.000
41	$C_{11}H_{15}N_5O_3S$	adenylthiomethylpentose	1.165	0.000
42	$C_{15}H_{22}O_8$	3,4,5-trimethoxyphenyl-1-*O*-glucoside	1.164	0.000
43	$C_{31}H_{61}O_{14}N$	3-hydroxypropyl palmitate glc-glucosamine	1.161	0.000
44	$C_{16}H_{18}O_9$	chlorogenic acid	1.161	0.000
45	$C_{17}H_{22}O_{10}$	1-*O*-glucosyl sinapate	1.160	0.000
46	$C_{22}H_{45}O_9P$	LysoPG 16:0	1.159	0.000
47	$C_{26}H_{52}NO_7P$	LysoPC 18:1	1.159	0.000
48	$C_{22}H_{46}NO_7P$	LysoPC 14:0	1.159	0.000
49	$C_6H_{13}NO_2$	6-deoxyfagomine	1.158	0.000
50	$C_9H_{10}O_5$	syringic acid	1.155	0.000
51	$C_8H_{15}NaO_2$	sodium valproate	1.157	0.000
52	$C_8H_7NO_2$	4-hydroxymandelonitrile	1.154	0.000
53	$C_5H_{14}N_4$	agmatine	1.154	0.000
54	$C_6H_{11}NO_4$	2-aminoadipicacid	1.153	0.000
55	$C_{24}H_{50}NO_7P$	LysoPC 16:0	1.153	0.000
56	$C_7H_6O_3$	4-hydroxybenzoic acid	1.152	0.000
57	$C_6H_{14}N_2O$	*N*-acetylputrescine	1.150	0.000
58	$C_{10}H_{12}N_4O_5$	arabinosylhypoxanthine	1.149	0.000
59	$C_{23}H_{26}O_{11}$	1'-*O*-(3,4-dihydroxyphenethyl)-*O*-caffeoyl-glucoside	1.144	0.000
60	$C_{27}H_{54}NO_7P$	LysoPC 19:1	1.144	0.000

61	$C_7H_6O_3$	protocatechualdehyde	1.142	0.000
62	$C_{20}H_{23}N_7O_7$	10-formyltetrahydrofuran	1.137	0.000
63	$C_{16}H_{20}O_{10}$	trihydroxycinnamoylquinic acid	1.136	0.000
64	$C_{21}H_{22}O_6$	dihydrocurcumin	1.136	0.000
65	$C_{28}H_{50}NO_7P$	propyl 2-(trimethylammonio) ethyl phosphate	1.133	0.000
66	$C_6H_{11}N_3O$	histidinol	1.132	0.000
67	$C_8H_7NO_3$	2-formamidobenzoate	1.132	0.000
68	$C_{21}H_{36}O_4$	1-α-linolenoyl-glycerol	1.131	0.000
69	$C_7H_6O_4$	2,4-dihydroxybenzoic acid	1.130	0.000
70	$C_{23}H_{46}NO_7P$	LysoPE 18:1	1.130	0.000
71	$C_{24}H_{38}O_4$	bis-(2-ethylhexyl)-phthalate	1.130	0.000
72	$C_8H_8O_4$	vanillic acid	1.129	0.000
73	$C_7H_6O_4$	gentisic acid	1.128	0.000
74	$C_7H_6O_4$	protocatechuic acid	1.124	0.000
75	$C_{23}H_{48}NO_7P$	LysoPC 15:0	1.123	0.000
76	$C_8H_{20}NO_6P$	choline alfoscerate	1.123	0.000
77	$C_{25}H_{52}NO_7P$	LysoPC 17:0	1.119	0.000
78	$C_{10}H_{14}N_5O_8P$	guanosine 5'-monophosphate	1.119	0.000
79	$C_{11}H_{14}NO_6$	nicotinic acid riboside	1.119	0.000
80	$C_8H_{11}NO_3$	pyridoxine	1.116	0.000
81	$C_{30}H_{48}O_4$	2α-hydroxyursolic acid	1.114	0.000
82	$C_{20}H_{20}O_5$	dihydrodemethoxy curcumin	1.112	0.001
83	$C_{34}H_{37}N_3O_6$	*N′*, *N′′*, *N′′′*-p-coumaroyl-cinnamoyl-caffeoyl spermidine	1.109	0.000
84	$C_{28}H_{56}NO_7P$	LysoPC 20:1	1.107	0.000
85	$C_{21}H_{38}O_4$	1-linoleoylglycerol	1.105	0.000
86	$C_{20}H_{28}O_3$	cafestol	1.105	0.000
87	$C_5H_{11}O_7P$	2-deoxyribose-1-phosphate	1.104	0.000
88	$C_{20}H_{28}O4$	pinusolidic acid	1.104	0.000
89	$C_6H_6N_2O$	nicotinamide	1.103	0.000
90	$C_5H_{13}NO$	choline	1.103	0.000
91	$C_{19}H_{28}O_6$	[4]-gingerdiol	1.102	0.000
92	$C_{28}H_{38}O_{14}$	ligraminol B	1.099	0.000
93	$C_5H_4N_4O_3$	uric acid	1.093	0.000
94	$C_{19}H_{24}O_3$	centrolobol	1.093	0.000
95	$C_{10}H_{13}N_5O_4$	adenosine	1.091	0.000
96	$C_{16}H_{18}O_9$	cryptochlorogenic acid	1.091	0.000
97	$C_{27}H_{48}O_9$	2-linoleoylglycerol-1-*O*-glucoside	1.090	0.000
98	$C_9H_{16}O_4$	eucommiol	1.089	0.000
99	C_8H_9N	*n*-benzylmethylene isomethylamine	1.089	0.000
100	$C_6H_7N_5$	1-methyladenine	1.087	0.000
101	$C_{12}H_{17}N_5O_5$	n,n-dimethylguanosine	1.085	0.000
102	$C_5H_5N_5$	adenine	1.079	0.000
103	$C_9H_{12}N_2O_6$	uridine	1.077	0.000
104	$C_6H_6N_4S$	6-methylmercaptopurine	1.069	0.000

105	$C_{22}H_{28}O_7$	5-hydroxy-7-(4-hydroxy-3,5-dimethoxyphenyl)-1-(4-hydroxy-3-methoxyphenyl) heptan-3-one	1.069	0.000
106	$C_4H_{12}N_2$	putrescine	1.066	0.000
107	$C_9H_{13}N_3O_5$	cytarabine	1.065	0.000
108	$C_8H_9NO_3$	4,5,6-trihydroxy-2-cyclohexen-1-ylideneacetonitrile	1.064	0.000
109	$C_9H_{13}N_2O_9P$	uridine 5'-monophosphate	1.061	0.000
110	$C_5H_5N_5O$	guanine	1.061	0.000
111	$C_{11}H_{14}O_5$	3,4'-dihydroxy-3',5'-dimethoxypropiophenone	1.061	0.000
112	$C_{21}H_{36}O_4$	2-*α*-linolenoyl-glycerol	1.057	0.000
113	$C_9H_{14}N_3O_8P$	cytidylic acid	1.053	0.000
114	$C_{10}H_{10}O_4$	ferulic acid	1.044	0.000
115	$C_{21}H_{28}N_7O_{17}P_3$	triphosphopyridine nucleotide	1.044	0.000
116	$C_5H_{15}ClNO_4P$	*O*-phosphocholine	1.040	0.000
117	$C_{14}H_{22}O$	2,6-di-tert-butylphenol	1.035	0.000
118	$C_{20}H_{30}O_3$	galanal A	1.031	0.000
119	$C_7H_6O_3$	salicylic acid	1.030	0.000
120	$C_5H_5N_5O$	isoguanine	1.016	0.000
121	$C_5H_5N_5$	2-aminopurine	1.014	0.000
122	$C_{11}H_{15}N_5O_4$	2'-*O*-methyladenosine	1.007	0.000
